# High-fat diet-induced and genetically inherited obesity differentially alters DNA methylation profile in the germline of adult male rats

**DOI:** 10.1186/s13148-020-00974-7

**Published:** 2020-11-19

**Authors:** Sharvari S. Deshpande, Harishankar Nemani, Gandhimathi Arumugam, Avinash Ravichandran, Nafisa H. Balasinor

**Affiliations:** 1 Department of Neuroendocrinology, ICMR-National Institute for Research in Reproductive Health, Jehangir Merwanji Street, Parel, Mumbai 400012 India; 2 National Institute of Nutrition Animal Facility, ICMR-National Institute of Nutrition, Jamai-Osmania PO, Hyderabad 500 007 India; 3 Genome Informatics Department, Genotypic Technologies Pvt. Ltd., #2/13, Balaji Complex, Poojari Layout, 80 Feet Road, R.M.V. 2nd stage, Bengaluru, India

**Keywords:** Obesity, Genetic, High-fat diet, DNA methylation, Spermatozoa, Embryo

## Abstract

**Background:**

Paternal obesity has been associated with reduced live birth rates. It could lead to inheritance of metabolic disturbances to the offspring through epigenetic mechanisms. However, obesity is a multifactorial disorder with genetic or environmental causes. Earlier we had demonstrated differential effects of high-fat diet-induced obesity (DIO) and genetically inherited obesity (GIO) on metabolic, hormonal profile, male fertility, and spermatogenesis using two rat models. The present study aimed to understand the effect of DIO and GIO on DNA methylation in male germline, and its subsequent effects on the resorbed (post-implantation embryo loss) and normal embryos. First, we assessed the DNA methylation enzymatic machinery in the testis by Real-Time PCR, followed global DNA methylation levels in spermatozoa and testicular cells by ELISA and flow cytometry, respectively. Further, we performed Methylation Sequencing in spermatozoa for both the groups. Sequencing data in spermatozoa from both the groups were validated using Pyrosequencing. Expression of the differentially methylated genes was assessed in the resorbed and normal embryos sired by the DIO group using Real-Time PCR for functional validation.

**Results:**

We noted a significant decrease in Dnmt transcript and global DNA methylation levels in the DIO group and an increase in the GIO group. Sequencing analysis showed 16,966 and 9113 differentially methylated regions in the spermatozoa of the DIO and GIO groups, respectively. Upon pathway analysis, we observed genes enriched in pathways involved in embryo growth and development namely Wnt, Hedgehog, TGF-beta, and Notch in spermatozoa for both the groups, the methylation status of which partially correlated with the gene expression pattern in resorbed and normal embryos sired by the DIO group.

**Conclusion:**

Our study reports the mechanism by which diet-induced and genetically inherited obesity causes differential effects on the DNA methylation in the male germline that could be due to a difference in the white adipose tissue accumulation. These differences could either lead to embryo loss or transmit obesity-related traits to the offspring in adult life.

## Background

Obesity is a chronic metabolic condition and is one of the leading risk factors for male sub-fertility or infertility [1]. Several reports have indicated that children born to obese fathers are at higher risk of developing metabolic syndrome in adult life [2, 3]. This evidence supports the notion that paternal obesity has a heritability component responsible for the transmission of obesity-related traits to the offspring, which is carried by the spermatozoa of obese fathers.

Epigenetic changes are heritable in nature and are brought about by DNA methylation, histone modifications and non-coding RNAs [4]. Several studies have shown that diet plays a critical role in modulating the epigenome [5]. Male rodents fed with a diet rich in fats show altered methylome profiles in the spermatozoa [6]. In addition, genome-wide studies in the spermatozoa of obese fathers show aberrant methylation and non-coding RNA expression profiles [7]. However, obesity is multifactorial in nature and could have the involvement of the environmental and/or genetic component [8]. The effects of these components individually on the epigenome of the male germline have not been explored earlier.

Animal-based obesity models are predominantly of two types (1) genetic models which are based on mutations or manipulations of one or more genes and (2) genetically healthy animals which are exposed to obesogenic environments, mainly high calorie-rich diets [9]. Our earlier studies have shown the differential effects of high-fat diet-induced (DIO) and genetically inherited obesity (GIO) on metabolic, biochemical and hormonal profiles, fertility parameters and spermatogenesis [10]. The study involved the use of WNIN/Ob mutant rats (Wistar origin) as the genetically inherited obese model and Wistar rat strain as the high-fat diet-induced obese model [10]. In WNIN/Ob rats, the mutation is present on chromosome 5 upstream of the leptin receptor. However, the gene harboring the mutation is yet to be characterized [11]. These mutant rats follow an autosomal incomplete dominant pattern as the mode of inheritance [12, 13]. The metabolic indices like insulin, cholesterol, triglycerides and leptin levels are high in this model with normal glucose levels [12]. Earlier, we reported differences in the metabolic parameters (body weights, adiposity index), biochemical parameters (serum glucose, cholesterol, triglyceride levels), hormonal profiles (serum testosterone, estradiol, LH, FSH, prolactin, leptin, insulin levels), fertility parameters (implantation losses, litter size, potency) and spermatogenesis (mitosis, meiosis, spermiogenesis) between the two types of obesity could be attributed to the difference in the white adipose tissue accumulation despite both the groups having similar body weights [10, 14]. This also explained the discrepancies in the literature on the effects of male obesity on sperm parameters, endocrine profile and fertility. Our fertility data reported that diet-induced obese group showed sub-fertility with increased embryo loss and reduced litter size whereas the genetically inherited obese group showed complete infertility due to reduced testosterone levels [10]. Quantitation of testicular cells based on ploidy and cell type–specific expression markers, to study the effect of obesity on spermatogenesis, demonstrated that both GIO and DIO altered mitosis that was evident from the increase in the spermatogonia and S-phase population. However, differential effects were noted on meiosis and spermiogenesis in the two groups [10]. Other significant and differential alterations noted in the testis of DIO and GIO groups include expression of hormone receptors, cytokines, markers of oxidative stress as well as cell cycle mediators [14]. We hypothesized that in addition to the differences in metabolic, hormonal, fertility and spermatogenic parameters observed earlier, there could be differences in the sperm epigenome in the DIO and GIO rats that could have led to differential effects post-fertilization. Thus, our present study aims to understand the effects of high-fat diet-induced and genetically inherited paternal obesity on DNA methylation in the male germline.

## Results

### Diet-induced and genetically inherited obesity differentially affect DNA methylating enzymatic machinery, global 5-mC density in different cell populations in the testis and global CpG methylation in spermatozoa

**Fig. 1:**
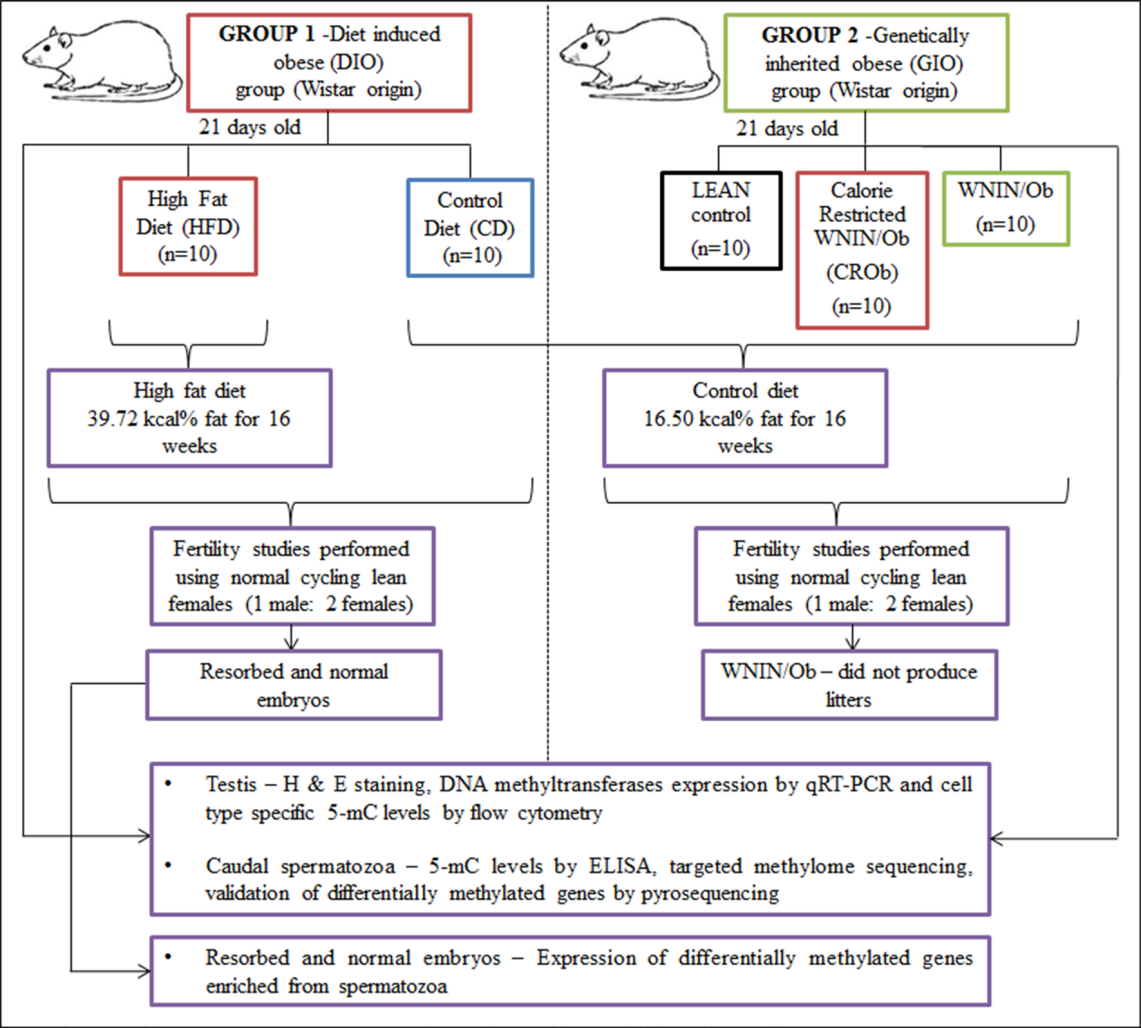
Schematic representation of the experimental design

The metabolic and phenotypic profiling of the animals were performed in our earlier study [10]. The schematic diagram of study design is depicted in Fig. 1.

**Fig. 2:**
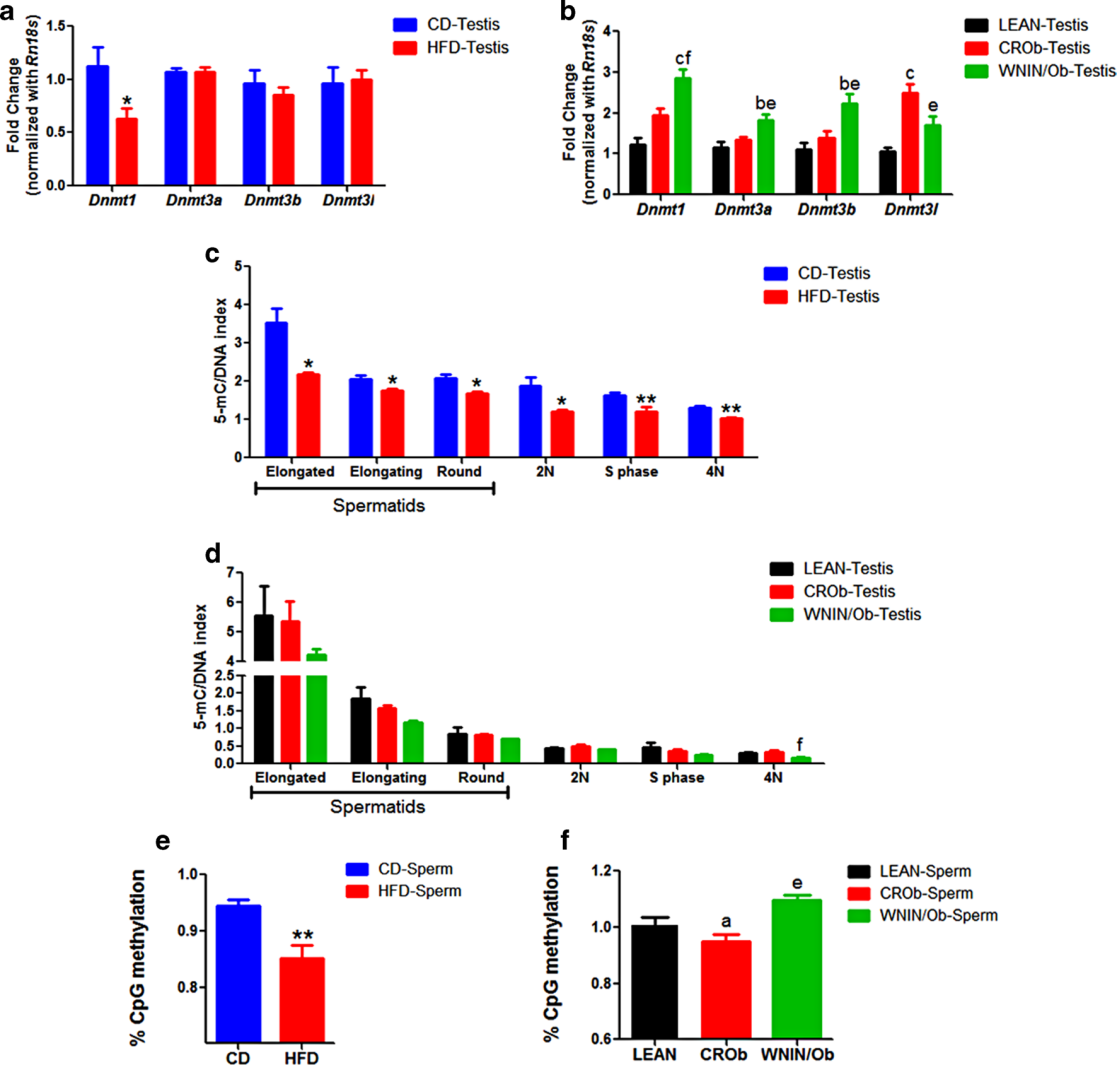
Effect of diet-induced (DIO) and genetically inherited obesity (GIO) on the transcript levels of DNA methylating enzymatic machinery, cell type-specific 5-methylcytosine (5-mC) density in the testis and global CpG methylation levels in spermatozoa. **a**, **b** Represent the transcript levels of DNA methyltransferases in the testis, **c**, **d** represent the 5-mC density in different testicular cell populations and **e**, **f** represent the percent CpG methylation levels in the spermatozoa of HFD group compared to CD group and WNIN/Ob group compared to CROb and LEAN groups, respectively. Data are expressed as mean ± S.E.M, N = 6 per group. Asterisks indicate significant differences compared with the CD group (*p < 0.05, **p < 0.01, ***p < 0.001); One-way ANOVA, Bonferroni multiple comparisons test: ^a^P < 0.05, ^b^P < 0.01, ^c^P < 0.001 compared with the LEAN group; ^e^P < 0.05, ^f^P < 0.01, ^g^P < 0.001 compared with the CROb group

To study the effect of high-fat diet-induced and genetically inherited obesity on DNA methylation in the male germline, we first assessed the DNA methyltransferases expression pattern in the testis. We observed a significant decrease in the *Dnmt1* transcript levels in the testis of the HFD group compared to the CD group (Fig. 2a). However, *Dnmt3a*, *Dnmt3b*, and *Dnmt3l* were found to be unaffected (Fig. 2a). On the contrary, we observed a significant increase in the *Dnmt1*, *Dnmt3a*, and *Dnmt3b* expression levels in the testis of the WNIN/Ob group compared to the CROb and LEAN groups, respectively (Fig. 2b). *Dnmt3l* expression, however, was down-regulated in the testis of the WNIN/Ob group when compared to the CROb group and was up-regulated in the CROb group as compared to the LEAN group (Fig. 2b). *Dnmt3l* transcript levels were unaffected in the testis of the WNIN/Ob group compared to the LEAN group (Fig. 2b).

To study whether *Dnmt* transcript levels correlate with the global DNA methylation levels in the testis, we further analyzed the global 5-methylcytosine (5-mC) levels in the different cell types of testis for both the groups. Knowing the fact that the different testicular cell types could be separated based on ploidy, we performed combined DNA methylation and the cell cycle analysis by flow cytometry. We observed a significant decrease in the 5-mC density in different testicular cell populations, mainly haploid population [elongated, elongating and round spermatids (RS)], 2 N population [spermatogonia, Sertoli cells, Leydig cells, peritubular myoid cells and secondary spermatocytes], S phase population [proliferating cells] and 4 N population [primary spermatocytes] in the testis of the HFD group compared to the CD group that correlated with the decrease in *Dnmt1* transcript levels in the testis observed in the HFD group as compared to the CD group (Fig. 2c). Interestingly, we did not observe any changes in the 5-mC density in the different testicular cell populations of the WNIN/Ob group compared to the CROb and LEAN groups, respectively (Fig. 2d). To substantiate that the obesity-induced epigenetic changes observed in the testis of DIO and GIO are not due to any anatomical defects, histological examination of testis was done. We did not observe any specific morphological changes in the testis of the two groups (Additional file 5: S1). Further as mentioned above, we had observed changes in the number of different testicular cell populations in the two groups [10]. Besides, data on methylation have been normalized to DNA content whereas data on gene expression to housekeeping genes to account for these differences.

To ascertain whether the DNA methylation changes in the spermatogenic cells translated in the mature spermatozoa of the two groups, we assessed global CpG methylation using ELISA. We noted a significant reduction in global CpG methylation in the spermatozoa of the HFD group compared to the CD group (Fig. 2e). In contrast, we observed a significant increase in the global CpG methylation in the spermatozoa of the WNIN/Ob group compared to the CROb group (Fig. 2f). However, upon calorie restriction, we found a significant reduction in the global CpG methylation in the spermatozoa of the CROb group compared to the LEAN group (Fig. 2f). No effect was observed on the global CpG methylation in the spermatozoa of the WNIN/Ob group compared to the LEAN group (Fig. 2f).

### DNA methylome analysis of spermatozoa of diet-induced and genetically inherited obese groups

To ascertain the effect of high-fat diet-induced and genetically inherited obesity on sperm epigenome, we performed targeted methylome sequencing in the spermatozoa of both the groups (DIO and GIO). Single base-pair resolution profile of 5-methylcytosine was generated by bisulfite sequencing of targeted captured DNA from the spermatozoa obtained from the DIO (CD and HFD) and GIO (LEAN, CROb and WNIN/Ob) groups, respectively. This generated an average of ~ 36 million raw reads in the CD and HFD groups and ~ 40 million raw reads in the LEAN, CROb and WNIN/Ob groups that upon further pre-processing yielded ~ 34 and ~ 36 million reads, respectively (Additional file 1: S1.2, Additional file 2: S2.1). Out of which ~ 18 and ~ 25 million reads uniquely aligned to the bisulfite-converted rat reference genome [Jul. 2014 (RGSC 6.0/rn6)] in the two groups (DIO and GIO), respectively (Additional file 1: S1.2, Additional file 2: S2.1). About 42% and 28% of the reads showed no alignment and about 7% and 9% of the reads showed ambiguous alignment to the reference genome in the CD and HFD groups, respectively (Additional file 1: S1.2, Additional file 2: S2.1). About 16%, 19% and 17% of the reads were unaligned and about 11%, 10% and 11% were unambiguous reads in the LEAN, CROb and WNIN/Ob groups, respectively (Additional file 1: S1.2, Additional file 2: S2.1). The total number of cytosines extracted from the unique reads was ~ 475 million in CD, ~ 593 million in HFD, ~ 739 million in LEAN, ~ 717 million in CROb and ~ 748 million in WNIN/Ob groups (Additional file 1: S1.2, Additional 2: S2.1). Of which, about ~ 17 and 21 million were methylated CpGs in CD and HFD groups while ~ 25, 24 and 25 million CpGs were methylated in LEAN, CROb and WNIN/Ob groups, respectively (Additional file 1: S1.2, Additional 2: S2.1). Interestingly, we observed high level of methylation (75–77%) in CpG context and low or negligible levels (0.4–1.4%) in the CHG and CHH context in all the five groups (Additional file 1: S1.2, Additional 2: S2.1, Additional 5: S5). We hereby report the evaluation of differentially methylated cytosines in the CpG context.

**Fig. 3:**
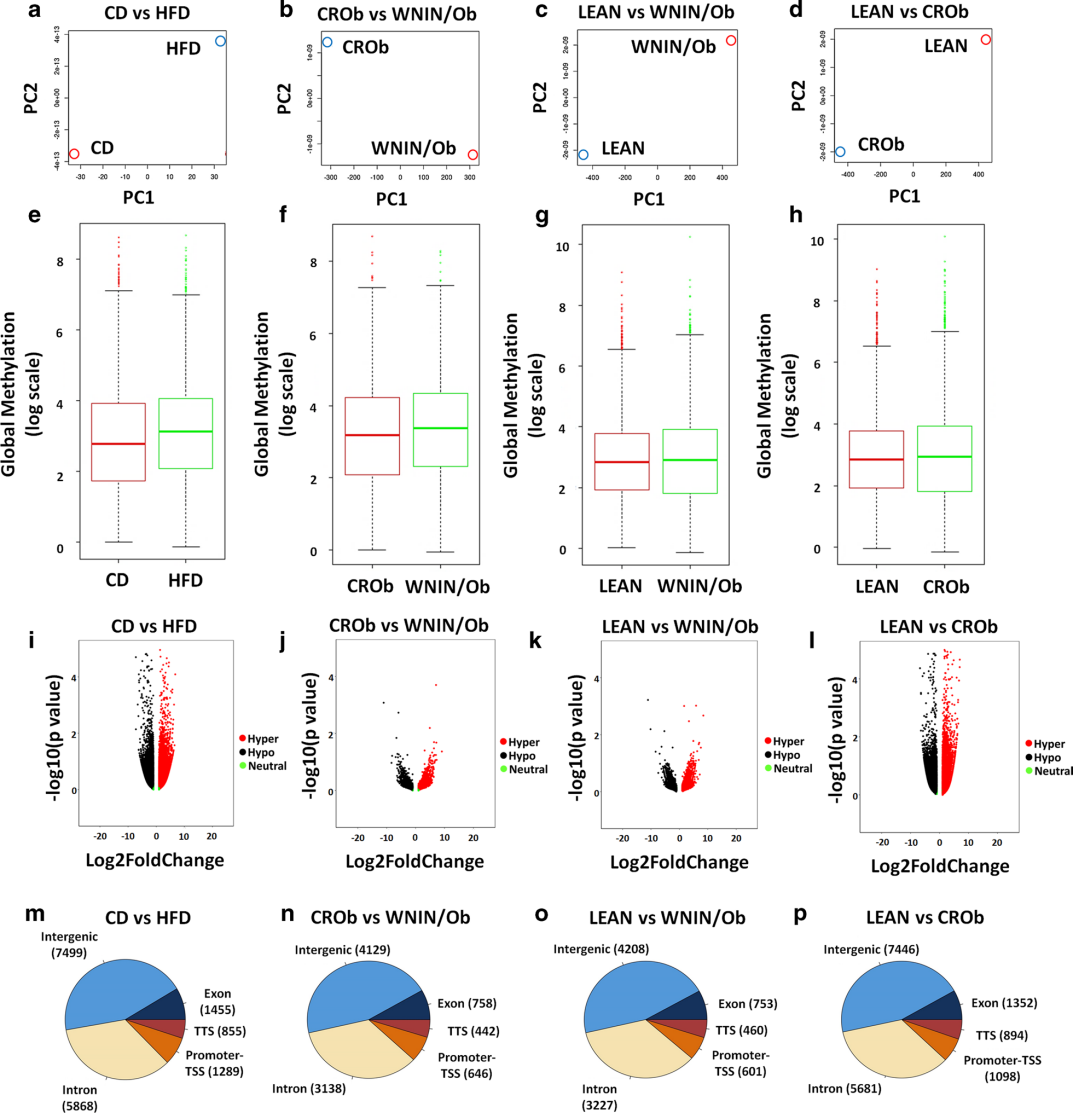
DNA methylome analysis of spermatozoa from diet-induced and genetically inherited obese groups. **a**–**d** represent principal component analysis (PCA) plots of DNA methylation profiles. Each colored dot represents a pooled DNA sample for one group. **e**–**h** Represent whisker box plots for the global methylation of differentially methylated CpGs. **i**–**l** represent volcano plots showing hypermethylated (red circles), hypomethylated (black circles) and neutral (green circles) CpGs. Each circle represents a single CpG site. **m**–**p** Pie charts representing the regional distribution of differentially methylated CpGs sites obtained in intergenic, exonic, TTS, promoter-TSS and intronic regions in spermatozoa of CD versus HFD, CROb versus WNIN/Ob, LEAN versus WNIN/Ob and LEAN versus CROb groups, respectively

Further, principal component analysis (PCA) on the methylation datasets for each group revealed distinct methylation profiles for each group (Fig. 3a–d). The overall global methylation levels in each group (CD vs. HFD; CROb vs. WNIN/Ob; LEAN vs. WNIN/Ob and LEAN vs. CROb) did not show any difference (Fig. 3e–h). In the HFD group compared to the CD group, methylation sequencing of spermatozoa identified 16,966 differentially methylated CpG sites, of which 9374 CpG sites were hypermethylated and 7592 CpG sites were hypomethylated (Additional file 1: S1.3, 1.4, 1.5). In the spermatozoa of the WNIN/Ob group compared to the CROb group, we observed 9113 differentially methylated CpG sites, of which 4854 CpG sites were hypermethylated and 4259 CpG sites were hypomethylated (Additional file 2: S2.2, S2.3, S2.4). 9249 differentially methylated regions were found in the spermatozoa of the WNIN/Ob group compared to the LEAN group, of which 4702 CpG sites were hypermethylated and 4547 CpG sites were hypomethylated (Additional file 3: S3.1, S3.2, S3.3). In the CROb group compared to the LEAN group, we noted 16,471 regions to be differentially methylated, of which 8600 CpG sites were hypermethylated and 7871 CpG sites were hypomethylated (Additional file 4: S4.1, S4.2, S4.3). Each differentially methylated CpG was visualized in the volcano plot of every group (Fig. 3i–l). The significantly hypermethylated CpGs are shown in red circles while hypomethylated CpGs are shown in black circles (Fig. 3i–l). Green circles represent the CpGs that were unchanged (Fig. 3i–l). It was interesting to note that there were similarities between the volcano plots of CD versus HFD, LEAN versus CROb, CROb versus WNIN/Ob and LEAN versus WNIN/Ob (Fig. 3i–l). Comparative analysis of CD versus HFD and CROb versus WNIN/Ob groups showed 3499 genes to be common between both the groups (Additional 5: S8). Interestingly, 10,543 genes were found to be unique to the HFD group compared to the CD group and 5927 genes were exclusive to WNIN/Ob group compared to the CROb group (Additional 5: S8). Comparative analysis of CROb versus WNIN/Ob, LEAN versus WNIN/Ob, and LEAN versus CROb groups showed 5927, 5830 and 10,218 genes unique to each group, respectively (Additional 5: S8). About 2540 genes were common to the three groups (Additional 5: S8). The majority of the differentially methylated CpG sites were mapped in the intergenic and intronic regions for all the four groups (Fig. 3m–p).

### Identification of unique pathways that are differentially altered in the spermatozoa of diet-induced and genetically inherited obese groups

To identify the molecular mechanisms of the differentially methylated genes enriched in the spermatozoa of diet-induced and genetically inherited obese groups, gene ontology and pathway analysis were performed using PANTHER (PANTHER 14.1) and KEGG databases. The majority of the differentially methylated genes were enriched in the binding and catalytic activity category for all the four groups (Additional file 1: S1.6, Additional 2: S2.5, Additional file 3: S3.4, Additional file 4: S4.4). Under biological processes, more than 1000 differentially methylated genes were enriched in the cellular process, metabolic process and biological regulation category (Additional file 1: S1.6, Additional 2: S2.5, Additional file 3: S3.4, Additional file 4: S4.4). Other enriched genes were found to be involved in response to stimulus, cellular component organization or biogenesis, localization, signaling, multicellular organismal process, developmental and immune system processes in all the four groups (Additional file 1: S1.6, Additional 2: S2.5, Additional file 3: S3.4, Additional file 4: S4.4).

Further, pathway analysis using the KEGG database showed enrichment of various significant pathways, namely, metabolic, pathways in cancer, PI3K-Akt, MAPK, thermogenesis, JAK-STAT, autophagy and insulin signaling in all the four groups (CD versus HFD, CROb versus WNIN/Ob, LEAN versus WNIN/Ob and LEAN versus CROb)(Additional file 1: 1.7, Additional 2: 2.6, Additional file 3: 3.5, Additional file 4: 4.5). Interestingly, some key signaling pathways involved in embryo development like Wnt, Hedgehog, TGF-beta (transforming growth factor-beta) and Notch were enriched (Additional file 1: 1.7, Additional 2: 2.6, Additional file 3: 3.5, Additional file 4: 4.5). The adjusted p value for each pathway is provided in the Additional file 1: S1.6 and S1.7, Additional 2: S2.5 and 2.6, Additional file 3: S3.4 and 3.5, Additional file 4: S4.4 and 4.5. The total number of differentially methylated genes enriched in each of the developmental pathways is as follows: Wnt pathway (58 – CD vs. HFD; 30 – CROb vs. WNIN/Ob), Hedgehog pathway (18—CD vs. HFD; 9—CROb vs. WNIN/Ob), TGF-beta pathway (35—CD vs. HFD; 30—CROb vs. WNIN/Ob) and Notch pathway (29—CD vs. HFD; 18—CROb vs. WNIN/Ob) (Additional file 1: S1.8-S1.11& Additional 2: S2.7-S2.10). Several genes of these pathways in the CD vs. HFD group overlapped with the CROb vs. WNIN/Ob group (Additional file 1: S1.8-S1.11 & Additional 2: S2.7-S2.10). However, the region and the position of CpG methylation change for these genes differed in both the groups for these genes (Additional file 1: S1.8-S1.11& Additional 2: S2.7-S2.10). To understand the effect of high-fat diet-induced and genetically inherited obesity on these developmental pathways in spermatozoa, we selected these genes for further validation. The list of genes enriched in each pathway for CD versus HFD and CROb versus WNIN/Ob and their involvement in the respective pathways are shown in Additional file 1: S1.8-S1.11& Additional 2: S2.7-S2.10 and Additional 5: S9-S12.

### Diet-induced and genetically inherited obesity differentially reprogram DNA methylation profile of molecular players of developmental pathways in spermatozoa

To ascertain the effect of high-fat diet-induced and genetically inherited obesity on developmental pathways in spermatozoa, few differentially methylated genes from Wnt, Hedgehog, TGF-beta and Notch signaling pathways that were exclusive to CD versus HFD and CROb versus WNIN/Ob groups were selected to validate the methylation changes by pyrosequencing (Tables 1, 2).

**Table 1:** Differentially methylated genes validated by pyrosequencing for CD versus HFD group

Pathway	Genes	Region	Chromosome number	Position
*CD versus HFD*				
Wnt	*Skp1*	Intergenic	10	37,590,812
	*Wnt9b*		10	91,777,080
	*Mapk10*		14	8,077,373
Hedgehog	*Spop*		10	83,217,039
TGF-beta	*Ppp2cb*		16	62,308,233
Notch	*Kat2b*		9	4,515,201

**Table 2:** Differentially methylated genes validated by pyrosequencing for CROb versus WNIN/Ob group

Pathway	Genes	Region	Chromosome number	Position	
*CROb versus WNIN/Ob*					
Wnt	*Sox17*	Intergenic	5		14,917,510
	*Wnt1*	Intergenic	7		140,463,854
Hedgehog	*Spopl*	Intergenic	3		496,642
	*Gli1*	Exon	7		70,622,579
TGF-beta	*Dcn*	Intergenic	7		38,710,172
	*Bmpr1b*	Intergenic	2		247,673,531
Notch	*Rbpj*	Promoter-TSS	14		4,679,285

**Fig. 4:**
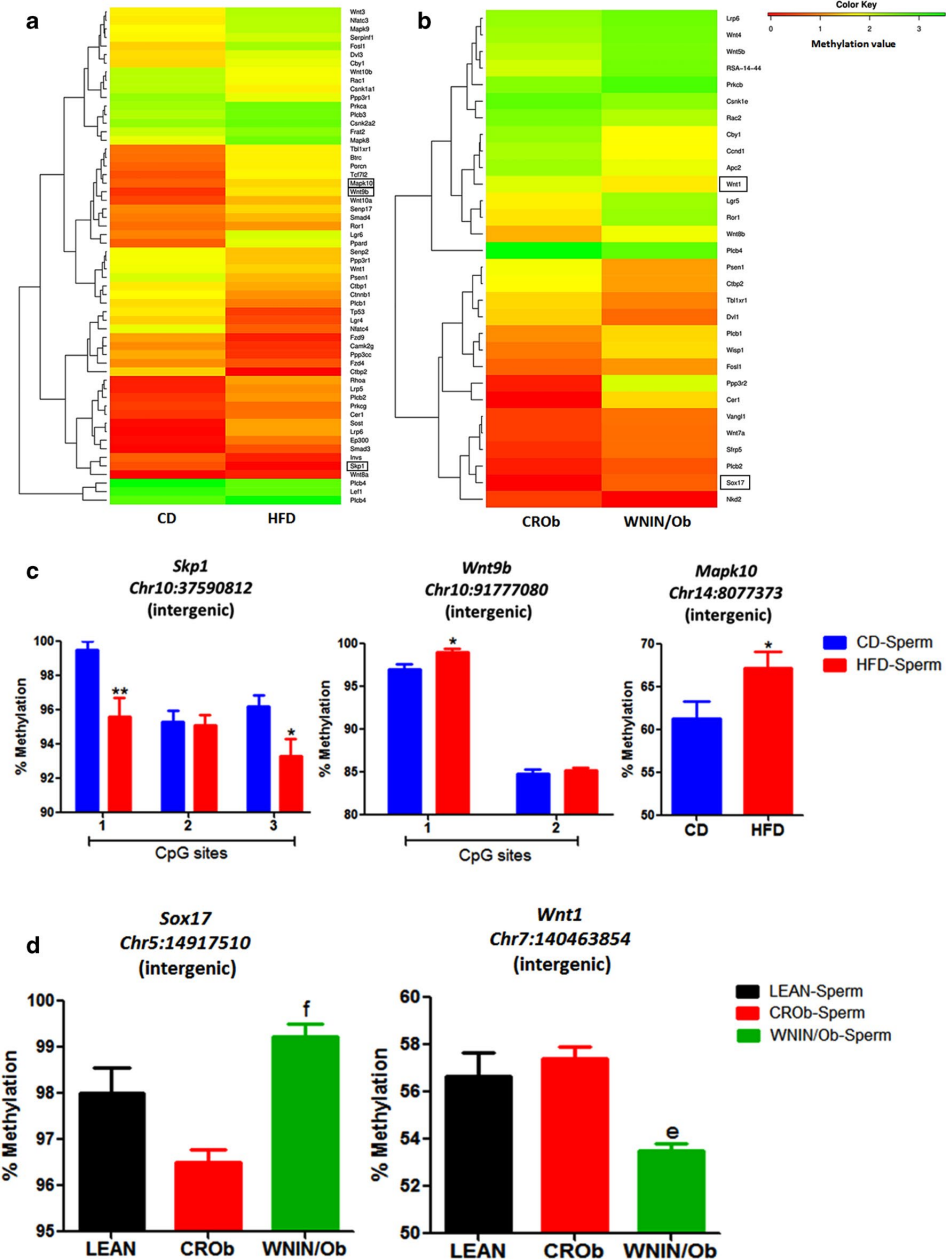
Effect of high-fat diet-induced and genetically inherited obesity on Wnt signaling pathway in spermatozoa. **a**, **b** Hierarchically clustered heatmap of the differentially methylated CpGs of genes associated with Wnt pathway. Black rectangle on the heatmap represents the genes validated by pyrosequencing. **c**, **d** Pyrosequencing validation of differentially methylated CpGs of genes involved in Wnt signaling in spermatozoa of DIO and GIO groups. Data are expressed as mean ± S.E.M, N = 3 samples pooled per group for methylation sequencing and N = 5 per group for pyrosequencing validation. Asterisks indicate significant differences compared with the CD group (*p < 0.05, **p < 0.01, ***p < 0.001). One-way ANOVA, Bonferroni multiple comparisons test: ^a^P < 0.05, ^b^P < 0.01, ^c^P < 0.001 compared with the LEAN group; ^e^P < 0.05^, f^P < 0.01, ^g^P < 0.001 compared with the CROb group

**Fig. 5:**
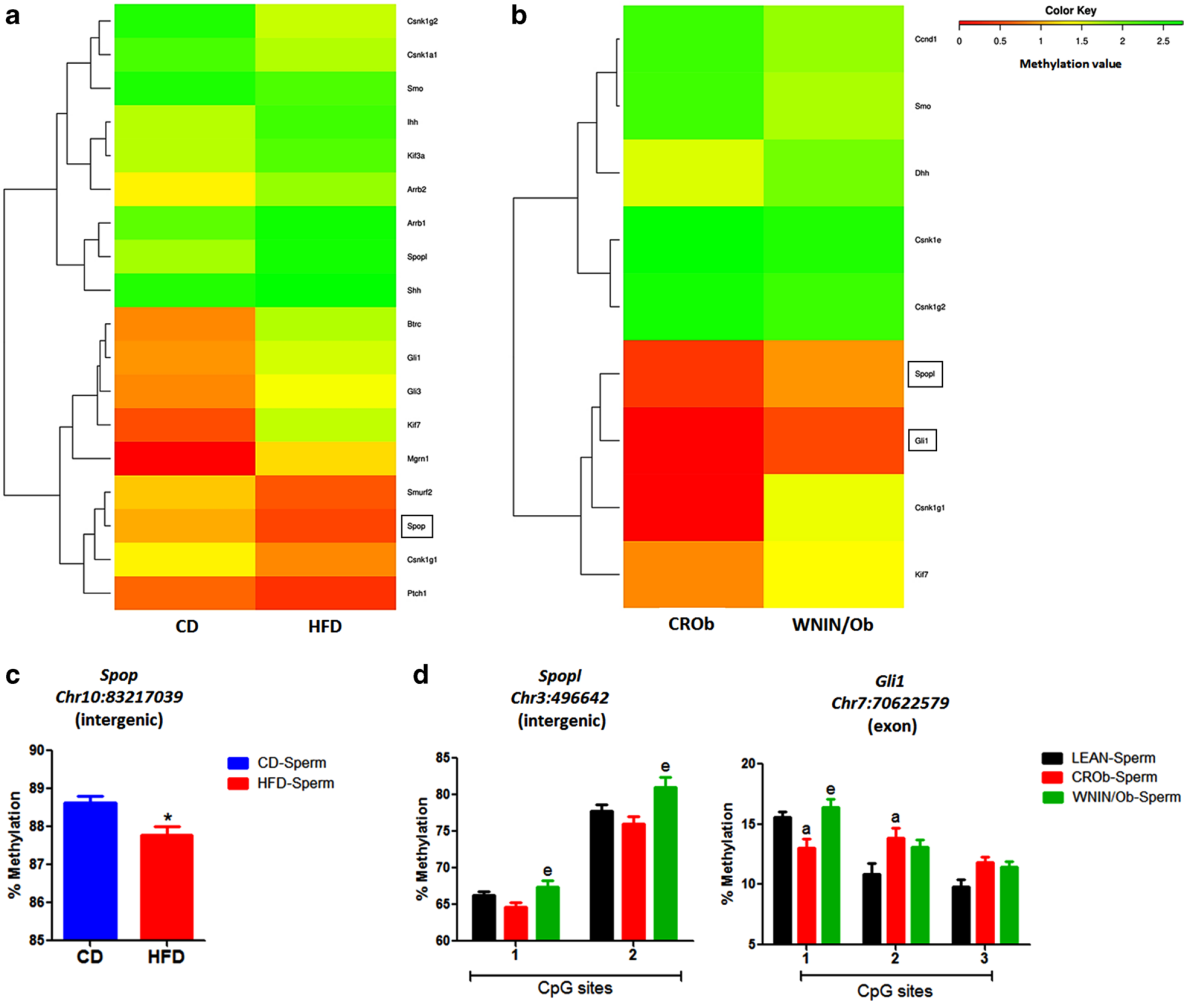
Effect of high-fat diet-induced and genetically inherited obesity on Hedgehog signaling pathway in spermatozoa. **a**, **b** Hierarchically clustered heatmap of the differentially methylated CpGs of genes associated with Hedgehog pathway. Black rectangle on the heatmap represents the genes validated by pyrosequencing. **c**, **d** Pyrosequencing validation of differentially methylated CpGs of genes involved in Hedgehog signaling in spermatozoa of DIO and GIO groups. Data are expressed as mean ± S.E.M, N = 3 samples pooled per group for methylation sequencing and N = 5 per group for pyrosequencing validation. Asterisks indicate significant differences compared with the CD group (*p < 0.05, **p < 0.01, ***p < 0.001). One-way ANOVA, Bonferroni multiple comparisons test: ^a^P < 0.05, ^b^P < 0.01, ^c^P < 0.001 compared with the LEAN group; ^e^P < 0.05, ^f^P < 0.01, ^g^P < 0.001 compared with the CROb group

**Fig. 6:**
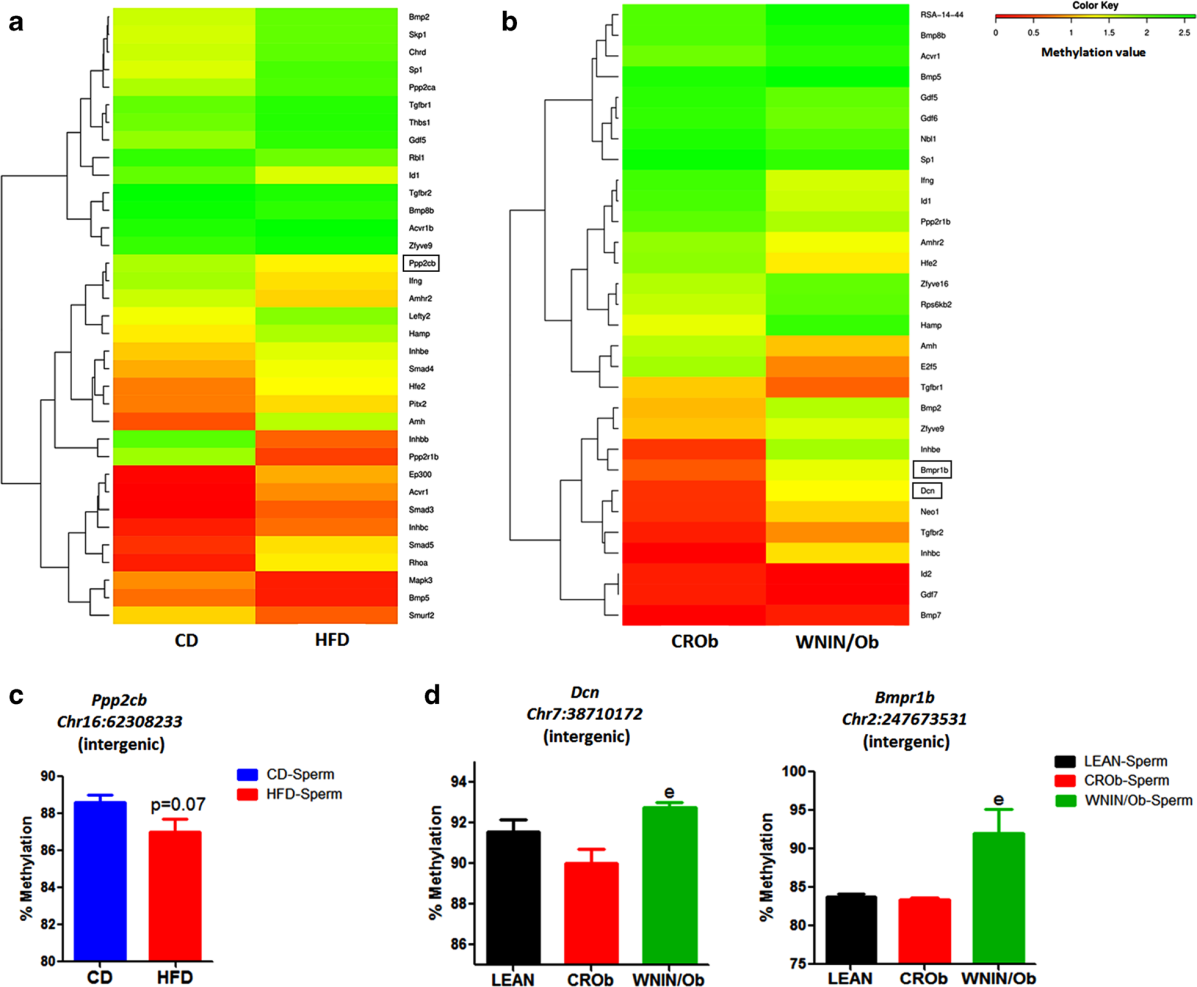
Effect of high-fat diet-induced and genetically inherited obesity on TGF-beta signaling pathway in spermatozoa. **a**, **b** Hierarchically clustered heatmap of the differentially methylated CpGs of genes associated with TGF-beta pathway. Black rectangle on the heatmap represents the genes validated by pyrosequencing. **c**, **d** Pyrosequencing validation of differentially methylated CpGs of genes involved in TGF-beta signaling in spermatozoa of DIO and GIO groups. Data are expressed as mean ± S.E.M, N = 3 samples pooled per group for methylation sequencing and N = 5 per group for pyrosequencing validation. Asterisks indicate significant differences compared with the CD group (*p < 0.05, **p < 0.01, ***p < 0.001). One-way ANOVA, Bonferroni multiple comparisons test: ^a^P < 0.05, ^b^P < 0.01, ^c^P < 0.001 compared with the LEAN group; ^e^P < 0.05, ^f^P < 0.01, ^g^P < 0.001 compared with the CROb group

**Fig. 7:**
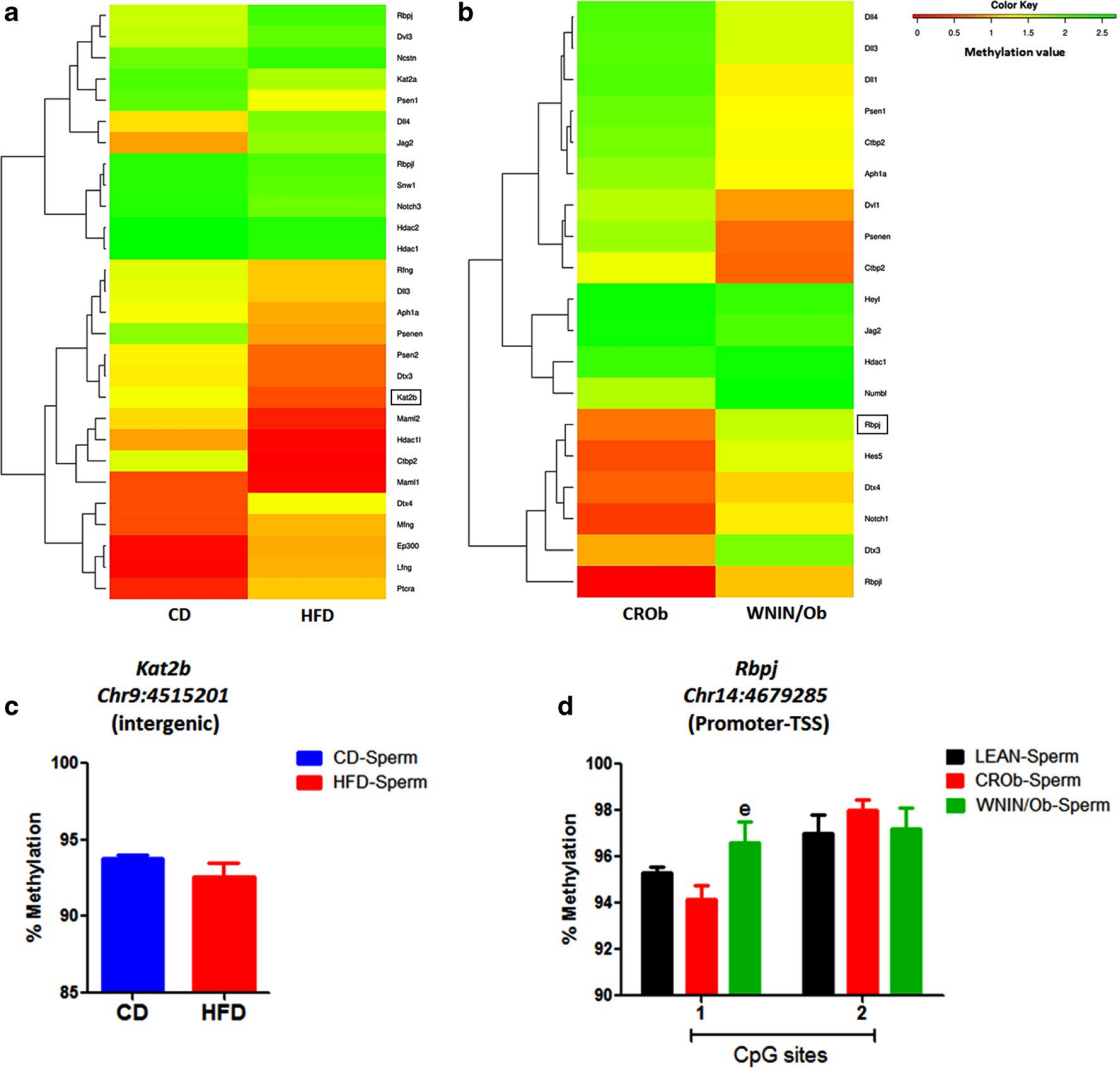
Effect of high-fat diet-induced and genetically inherited obesity on Notch signaling pathway in spermatozoa. **a**, **b** Hierarchically clustered heatmap of the differentially methylated CpGs of genes associated with Notch pathway. Black rectangle on the heatmap represents the genes validated by pyrosequencing. **c**, **d** Pyrosequencing validation of differentially methylated CpGs of genes involved in Notch signaling in spermatozoa of DIO and GIO groups. Data are expressed as mean ± S.E.M, N = 3 samples pooled per group for methylation sequencing and N = 5 per group for pyrosequencing validation. Asterisks indicate significant differences compared with the CD group (*p < 0.05, **p < 0.01, ***p < 0.001). One-way ANOVA, Bonferroni multiple comparisons test: ^a^P < 0.05, ^b^P < 0.01, ^c^P < 0.001 compared with the LEAN group; ^e^P < 0.05, ^f^P < 0.01, ^g^P < 0.001 compared with the CROb group

We first generated a hierarchically clustered heatmap based on the differences in the methylation value of CpGs for the genes associated with each developmental pathway for CD versus HFD (Figs. 4a, 5a, 6a, 7a) and CROb versus WNIN/Ob groups (Figs. 4b, 5b, 6b, 7b). The heatmap in every pathway in every group revealed differential changes in the methylation pattern (hypermethylation and hypomethylation) of developmental genes in spermatozoa (Figs. 4a, 5a, 6a, 7a) (Figs. 4b, 5b, 6b, 7b). 13 differentially methylated genes were randomly selected from Wnt, Hedgehog, TGF-beta and Notch signaling pathways from both the CD versus HFD and CROb versus WNIN/Ob datasets for pyrosequencing-based validation (Tables 1, 2, Additional file 1: S1.8-S1.11, Additional 2: S2.7-S2.10). Among these, 6 genes (*Skp1*, *Wnt9b*, *Mapk10*, *Spop*, *Ppp2cb*, *Kat2b*) were found to be differentially methylated in the spermatozoa of the HFD group compared to the CD group (Figs. 4c, 5c, 6c, 7c) and 7 genes (*Sox17*, *Wnt1*, *Spopl*, *Gli1*, *Dcn*, *Bmpr1b*, *Rbpj*) showed differential methylation pattern in the spermatozoa of the WNIN/Ob group compared to the CROb group (Figs. 4d, 5d, 6d, 7d). The differentially methylated CpG sites of all the selected genes, acquired by high throughput sequencing, represented in the form of heatmap, correlated with the pyrosequencing data in the spermatozoa of the HFD group compared to the CD group and of the WNIN/Ob group compared to the CROb and LEAN group, respectively (Figs. 4a, 5a, 6a, 7a) (Figs. 4b, 5b, 6b, 7b). The specific genes enriched in the CD versus HFD group for each pathway were also validated in the CROb versus WNIN/Ob group and vice versa. We did not observe any significant changes in the spermatozoa suggesting these genes were exclusive to their respective groups and thus additionally validated the NGS data (Data not shown).

### Methylation defects in spermatozoa are partially translated in the resorbed and normal embryos sired by high-fat diet-induced obese male rats

Our earlier studies had demonstrated that high-fat diet-induced obese male rats showed increased pre- and post-implantation loss and a significant reduction in the litter size, while the genetically inherited obese male rats were infertile [10]. In the present study, we observed methylation defects in the genes of developmental importance in the spermatozoa of high-fat diet-induced obese male rats. Therefore, we examined the gene expression patterns of those differentially methylated genes in the resorbed and normal embryos sired by both the HFD and CD groups, respectively.

**Fig. 8:**
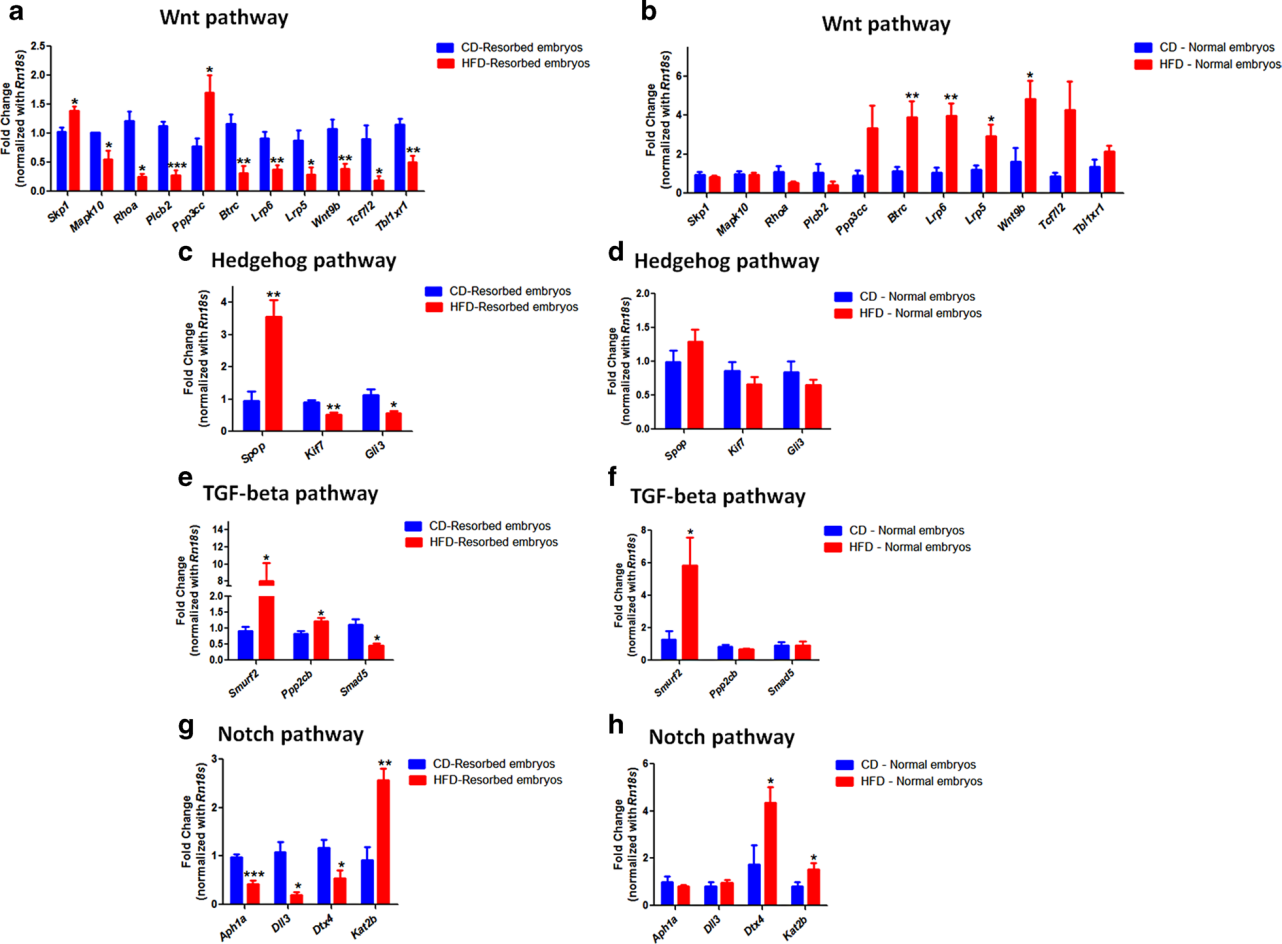
Effect of high-fat diet-induced and genetically inherited obesity on expression of genes associated with **a**, **b** Wnt, **c**, **d** Hedgehog, **e**, **f** TGF-beta and **g**, **h** Notch signaling pathways in resorbed and normal embryos. Data are expressed as mean ± S.E.M, N = 6 per group. Asterisks indicate significant differences compared with the CD group (*p < 0.05, **p < 0.01, ***p < 0.001)

In the Wnt signaling pathway, the expressions of *Skp1* and *Ppp3cc* were upregulated, whereas the expressions of *Mapk10*, *Rhoa*, *Btrc*, *Lrp6*, *Lrp5*, *Wnt9b*, *Tcf7l2* and *Tbl1xr1* were downregulated in the resorbed embryos sired by the HFD group compared to the CD group (Fig. 8a). On the contrary, *Skp1*, *Mapk10*, *Rhoa*, *Plcb2*, *Ppp3cc*, *Tcf7l2* and *Tbl1xr1* transcript levels were unaffected whereas *Btrc*, *Lrp6*, *Lrp5* and *Wnt9b* were upregulated in the normal embryos sired by the HFD group compared to the CD group (Fig. 8b). In the Hedgehog signaling pathway, *Spop* transcript levels were upregulated, while that of *Kif7* and *Gli3* transcript levels were downregulated in the resorbed embryos of the HFD group compared to the CD group (Fig. 8c). However, in the normal embryos, *Spop*, *Kif7* and *Gli3* transcript levels were unaltered in the HFD group compared to the CD group (Fig. 8d). In the TGF-beta signaling pathway, we observed significant upregulation of *Smurf2* and *Ppp2cb* transcript levels and downregulation of *Smad5* levels in the resorbed embryos of the HFD group compared to the CD group (Fig. 8e). Conversely, *Ppp2cb* and *Smad5* transcript levels were unchanged, whereas *Smurf2* was upregulated in the normal embryos of the HFD group compared to the CD group (Fig. 8f). Similarly, in the Notch signaling pathway, we noted a downregulation of *Aph1a*, *Dll3*, and *Dtx4* expression levels and the upregulation of *Kat2b* in the resorbed embryos of the HFD group compared to the CD group (Fig. 8g). On the other hand, *Aph1a* and *Dll3* transcript levels were unaltered, whereas *Dtx4* and *Kat2b* were upregulated in the normal embryos sired by the HFD group compared to the CD group (Fig. 8h). Interestingly, the expression patterns of all the genes in the different pathways in the resorbed embryos correlated with the methylation pattern in the spermatozoa of the HFD group compared to the CD group, which was not the case with the normal embryos sired by the HFD group compared to the CD group (Figs. 4, 5, 6, 7, 8).

Interestingly, we also noted the expression of a few genes in the resorbed embryos that did not correlate with the methylation pattern in the spermatozoa. For example, in the Wnt pathway, we observed hypermethylation of *Ppard*, *Mapk8*, *Prkcb*, and *Fosl1* in the spermatozoa of the HFD group compared to the CD group (Additional 5: S13). However, expression of these genes was significantly upregulated in the resorbed embryos of the HFD group compared to the CD group, whereas, *Fosl1* showed a reduced expression pattern but the change was not significant (Additional 5: S13). Similarly, genes in other pathways also showed a similar trend (Additional 5: S13). Furthermore, gene expression in the normal embryos sired by the HFD group also did not correlate with the methylation changes in the spermatozoa (Additional 5: S13).

## Discussion

Here, we provide evidence that high-fat diet-induced and genetically inherited obesity differentially alters the DNA methylome in the spermatozoa and affects the pathways involved in embryo development. DNA methylation is a key epigenetic contributor responsible for gene silencing with the help of the enzymes known as DNA methyltransferases (Dnmts) [15]. Therefore, we first assessed the *Dnmt* transcript levels in the testis of both the groups. We observed down-regulation of the *Dnmt1* transcript levels of the DIO group, up-regulation of *Dnmt1*, *Dnmt3a*, and *Dnmt3b* and down-regulation of *Dnmt3l* levels in the GIO group in the testis. *Dnmt1* is a maintenance methyltransferase that is associated with replication foci and maintains DNA methylation after replication, and localized in all the male germ cells [16, 17]. On the other hand, *Dnmt3a* and *Dnmt3b* are involved in catalyzing the active de novo methylation process on the DNA [16]. *Dnmt3l* lacks catalytic activity but is involved in the regulation of DNA methylation through its association with Dnmt3a and Dnmt3b [18]. Several studies have shown the importance of de novo methyltransferases in regulation of spermatogenesis. De novo methylation in the germ cells could be involved in genomic imprinting, germ cell-specific methylation or post-DNA repair methylation [19]. In the rat testes, Dnmt3a and Dnmt3b are both localized in the spermatogonial cells. Dnmt3a is also expressed in spermatocytes whereas Dnmt3l is localized in the nuclei of round spermatids, in the head region of the elongating spermatids, and in the peritubular cells of the adult rodent testis [20, 21]. *Dnmt* levels are mainly correlated with the global 5-mC levels. In the present study, we noted a significant reduction in global 5-mC density in different testicular cell populations that correlated with the *Dnmt1* expression levels, which were downregulated in the DIO group. Interestingly, Fullston et al. 2013 also demonstrated that paternal high-fat diet-induced obesity decreases global 5-mC levels in the testes that corroborates with our findings [2]. However, in the GIO group, no change was observed in the 5-mC density in the testis, despite the *Dnmts* being upregulated. Histological examination of the testis in the two groups also confirmed that the epigenetic changes are not due to the differences in testicular morphology but rather due to the high-fat diet-induced and genetically inherited obesity. Our histological findings corroborate with the findings of previous published studies in which no morphological defects were observed in the testis of DIO and GIO groups [13, 22]. Our previous studies have shown a significant increase in the spermatogonial cell number in both the DIO and GIO groups and a significant decrease in the primary spermatocyte number in the GIO group [10]. In the present study, we did not observe any changes in the *Dnmt3a* and *Dnmt3b* expression levels in the testis of the DIO group whereas in GIO group we noted an increase in the same. This suggests that the changes in the levels are not due to the alterations in cellular composition or number in the testis but rather due to the difference in the physiological changes in the two obese rat models.

Moreover, we also assessed the CpG methylation levels in the spermatozoa of both the groups. In the DIO group, the decrease in CpG methylation levels in spermatozoa correlated with the decrease in *Dnmt1* levels in the testis whereas an increase in CpG methylation corroborated with the increase in the *Dnmt* levels in the testis in the GIO group. Changes in Dnmts and/or global 5-mC levels due to two different types of obesities in the male germline could be attributed to a variety of factors like high oxidative stress, pro-inflammatory cytokines and methyl donor imbalance (for example, S-Adenosyl Methionine (SAM)) as well as endocrine dysfunction. Changes in these factors have been shown to be associated with DNA methylation defects [23–26]. Another plausible reason for the differential changes in the Dnmt and global DNA methylation levels could be due to the difference in white adipose tissue accumulation in the two groups. As in the GIO group, we have observed that upon calorie restriction, Dnmt levels in the testis were restored in the CROb group and were comparable to that of the LEAN controls. Also, another mechanism could be due to the loss of equilibrium between DNA methylation and demethylation. This balance between the two pathways is critical for the normal functioning of the germ cells [27]. We have also noted distinct changes in ten-eleven translocation (Tets) expression and 5-hydroxymethylcytosine (5-hmC) levels in the testis of both the groups (unpublished observations) suggesting an imbalance in the enzymatic machinery of 5-mC and 5-hmC. Interestingly, upon calorie restriction, we noted a decrease in CpG methylation levels in the spermatozoa of the CROb group compared to the LEAN group. This could probably be a combinatorial effect of genetic factors and calorie restriction.

Since we observed differential changes in the global DNA methylation levels in the spermatozoa of DIO and GIO groups, we next performed high-throughput methylation sequencing on the spermatozoa of the two groups to acquire a genome-wide picture. Sequencing data analysis revealed that spermatozoa harbor both CpG and non-CpG methylation. However, the percentage of methylated cytosines in each sequence context, namely, CpG, CHG and CHH in the control sperm is ~ 77%, ~ 0.5–1.4% and ~ 0.4–1.3%, respectively. Our data corroborated with the findings of Tomizawa et al. 2011 [28]. Hence, further genome-wide methylation data analysis was performed to specifically assess CpG methylation. We identified a total of 16,966, 9113, 9249 and 16,471 differentially methylated CpG sites in the spermatozoa of the HFD group compared to the CD group, WNIN/Ob group compared to the CROb group, WNIN/Ob group compared to the LEAN group and CROb group compared to the LEAN group respectively. Interestingly, the global methylation levels of the total CpGs analyzed from the sequencing data did not correlate with the ELISA-based CpG methylation levels in the spermatozoa for each group. This could probably be due to difference in the techniques and usage of pooled genomic DNA samples for sequencing. Further, comparative analysis between the DIO and GIO groups showed the presence of both unique and overlapping differentially methylated regions/genes in the spermatozoa. Our previous studies have shown significant changes in the white adipose tissue accumulation between the two groups despite their body weights being similar. This could have led to the differences in the biochemical, endocrine and fertility profile as well as spermatogenesis observed between the DIO and GIO groups [10, 14]. From the literature, we understand that any changes in metabolic and hormonal profile could alter the epigenetic mechanisms [2, 6]. Obesity is also known to be associated with increased oxidative stress [29]. Thus, we speculate that the methylome differences in spermatozoa could be a combination of these various altered parameters. Also, presence of unique regions/genes in spermatozoa is an indication of the exclusive effects of high-fat diet-induced obesity and genetically inherited obesity. In addition, the majority of the CpG sites were found to be differentially methylated in the intergenic (~ 45%) and intronic (~ 34%) regions in the spermatozoa. Both intergenic and intronic regions are associated with enhancers [30–32]. Interestingly, several studies have shown that methylation at intergenic and intronic regions where the enhancers reside regulate gene expression [32–35]. Aberrant methylation in these regions has been shown to be associated with aberrant gene expression in breast cancer [30]. Genome-wide methylome study by de Castro Barbosa et al. 2015 reported 109 differentially methylated genes in the spermatozoa of rats fed with high-fat diet [6]. Comparison with our DIO dataset showed 44 differentially methylated genes to be common between the two studies (Additional file 1: S1.15). Further, pathway analysis of the differentially methylated genes of all the four groups showed the involvement of these genes in numerous pathways, namely, metabolic, pathways in cancer, PI3K-Akt, MAPK, thermogenesis, JAK-STAT, autophagy, insulin, etc. Dysregulation of these fundamental pathways has been shown to be associated with obesity [2, 3, 36–43]. It is interesting to note that the genes/pathways enriched in the spermatozoa of both the DIO and GIO groups in the present study has similarity to phenotypic or physiological changes, namely, metabolic, adiposity, endocrine, spermatogenesis, oxidative stress, inflammation, apoptosis and cell cycle observed in the two groups in our previous studies [10, 14]. These observations establish a causal link between the sperm methylome and phenotypic or physiological changes induced due to DIO and GIO. Since some of these pathways have been explored before with respect to obesity, we identified a few other pathways of developmental importance, namely Wnt, Hedgehog, TGF-beta, and Notch that have never been investigated in the spermatozoa and linked to the embryo loss that had been earlier observed in our obese models [10].

Wnt signaling plays a pivotal role in both embryonic development and adult physiology and is involved in body axis patterning, cell fate specification, cell migration and proliferation [44, 45]. Another developmental pathway, Hedgehog is required for the development of intercellular communication, organogenesis, regeneration and homeostasis [6, 47]. Transforming growth factor-beta (TFG-beta) signaling regulates various cellular functions namely cell proliferation, differentiation, migration, adhesion and apoptosis [48, 49]. Notch proteins are single-pass receptors that are activated by delta and Jagged/Serrate families of membrane-bound ligands [50]. The notch pathway regulates various cellular processes like proliferation, differentiation, cell fate and apoptosis [51]. Several knockout studies have shown that loss of upstream and downstream molecular players of these developmental pathways are associated with embryo lethality, suggesting their critical role during embryo development [52–58]. It was interesting to note that almost all the differentially methylated genes in these developmental pathways were unique to the DIO and GIO groups as evident from the methylation pattern depicted in the heatmap. Validation of the methylation status of all the selected genes associated with these pathways in the spermatozoa by pyrosequencing confirmed the high-throughput sequencing results for DIO and GIO groups.

It is well evident in the literature that the sperm epigenome is involved in embryogenesis and offspring health [59]. Earlier studies in our laboratory have shown that paternal obesity leads to embryo loss and caused significant reduction in litter size [10]. Therefore, we analyzed the methylation status of developmental genes in spermatozoa and their expression in resorbed and normal embryos sired by the DIO model. The functional validation of gene expression of differentially methylated genes from these pathways was carried out in resorbed and normal embryos sired by the DIO group only. Expression of the genes associated with developmental pathways in the resorbed embryos but not the normal embryos correlated with the methylation pattern in spermatozoa of the DIO group. Overall, these results indicate that DIO and GIO differentially altered the developmental pathways in spermatozoa and methylation defects in these pathways in the spermatozoa could have led to aberrant expression of these genes in the embryos leading to embryo loss in the DIO group. The functional validation of the pathways in the embryos of the GIO group was not possible, as the WNIN/Ob group was infertile and did not produce any litters [10].

On the other hand, in normal embryos, we did not observe any correlation of the gene expression to that of sperm methylome. This could be due to the presence of ‘epigenetically fit’ spermatozoa in the sperm pool. Our previous studies on tamoxifen treatment in adult male rats had reported that not all spermatozoa in the treated group had abnormal methylation at imprinted gene loci *Igf2*-*H19* ICR [60]. This suggests that the normal embryos could be a result of fertilization of the oocyte with ‘epigenetically fit’ spermatozoa, whereas resorbed embryos could be a result of fertilization with ‘epigenetically unfit’ spermatozoa.

In resorbed embryos, expression of few of the differentially methylated genes did not correlate to their methylation pattern in the spermatozoa of the DIO group. This indicates that differential gametic methylation is either partially translated or not persistent in the embryos. This could be due to either post-fertilization genome-wide reprogramming or changes in other regulatory mechanisms like histone modifications (unpublished observations) and non-coding RNAs [61, 62]. These epigenetic changes may modify embryonic development during genome-wide reprogramming that occurs after fertilization or could persist to alter the gene expression and accumulate in the key tissues to show its effect at adulthood. In the Methyl Seq data, we also observed genes of several DNA modifying enzymes (Dnmts and Tets) and histone modifying enzymes [histone acetyltransferases (HATs), histone deacetylases (HDACs), histone demethylases (HDMs) and histone methyltransferases (HMTs)] to be differentially methylated in spermatozoa for both the groups. In addition, another reason for the differences in the gene expression patterns could be sex specific [63], as the sex of the embryos was not assessed in the present study.

In embryos, non-CpG methylation is prevalent and is mainly mediated by the de novo methyltransferases. Non-CpG methylation is critical for cellular development and differentiation [64]. It is possible that the changes in expression pattern of the genes associated with developmental pathways in the resorbed and normal embryos could be contributed by non-CpG methylation changes. Since the methylation status of the de novo methyltransferases are altered in spermatozoa of both the DIO and GIO groups, the non-CpG methylation in these embryos could be altered. Taken together, these evidences indicate that paternal obesity could be responsible for changes in the epigenome modifying enzymatic machinery in the spermatozoa that could alter the genome-wide reprogramming after fertilization thus leading to either embryo loss or cause defects at adulthood in the DIO group.

## Conclusion

Taken together, our study provides a mechanism through which diet-induced and genetically inherited obesity differentially reprograms the DNA methylome profile in the male germline. We provide a comprehensive picture of the genes and the pathways that are common and uniquely altered in the spermatozoa of diet-induced and genetically inherited obesity. We show that paternal diet-induced obesity could partially transmit the defective epigenetic signatures of developmental importance via sperm to the embryo thereby leading to embryo loss. We also show that calorie restriction of the genetically obese male rats could bring about partial changes in the germline by modulating the epigenome leading to partial restoration of the fertility in the WNIN/Ob group [10]. The differential changes in the methylation profiles in the germline between the DIO and GIO groups could be due to the difference in the white adipose tissue accumulation, which we had reported, in our previous study [10]. Knowledge of the altered genes or pathways in the spermatozoa due to diet-induced and genetically inherited obesity will further our understanding of the role of paternal contribution in the transmission of defective epigenetic signatures, leading to either embryo loss or transgenerational inheritance of deleterious traits to the offspring at adulthood as reported by other studies [2, 3, 6].

## Materials and methods

### Animals

High-fat diet-induced obese (DIO) Wistar male rat model was developed at the ICMR-National Institute for Research in Reproductive Health (ICMR-NIRRH), Mumbai, India. A study on genetically inherited obese (GIO) male rat model of Wistar origin was performed at ICMR-National Institute of Nutrition (ICMR-NIN), Hyderabad. All animals were maintained under controlled temperature (22 °C ± 1 °C) and humidity conditions with a 12:12 h light: darkness cycle. Prior approval for the use of animals for the experimental study was taken from the Institutional Animal Ethics Committee of both the Institutes.

### Group 1. Diet-induced obese (DIO) group consisted of 2 sub-groups (n = 10 animals per group)

At 21 days of age, randomly bred Wistar male rats were maintained either on a high-fat diet (HFD) containing 39.72 kcal% fat, 27.92 kcal% protein and 32.35 kcal% carbohydrate or on control diet (CD) containing 16.50 kcal% fat, 23.83 kcal% protein, 59.65 kcal% carbohydrate for 16 weeks. Both the diets were formulated at National Institute of Nutrition Animal Facility, ICMR-NIN, Hyderabad. Food and water were available ad libitum. Body weights of each animal were monitored once a week for 16 weeks as reported in our earlier study [10]. The detailed composition of the diets is provided in Additional file 1: S1.1.

### Group 2. Genetically inherited obese (GIO) group consisted of 3 sub-groups (n = 10 animals per group)

21-day-old Wistar origin WNIN/Ob (homozygous mutant), CROb (calorie-restricted WNIN/Ob—homozygous mutant) and LEAN (homozygous wild type) male rats were used for the study. Male rats age- and body weight-matched to the DIO group were used. Calorie restriction was carried out for WNIN/Ob group so as to rule out the confounding factors of the mutation and report obesity-induced effects. Calorie restriction was achieved by pair feeding, such that the amount of control diet consumed by the LEAN control male rats was calculated and the same amount was fed to 35 days old WNIN/Ob male rats [10, 65, 66]. The calorie restriction experiments on GIO group were performed at National Institute of Nutrition Animal Facility, ICMR-NIN, Hyderabad. The age (17 weeks old) and body weights of the GIO group were matched to the DIO group for the experimental study. All animals in the GIO group were maintained on control diet (CD) containing 16.50 kcal% fat, 23.83 kcal% protein and 59.65 kcal% carbohydrate. Food and water were available ad libitum.

### Mating studies and sample collection

All the male rats (17 weeks old) were cohabited with normal cycling lean female rats (n = 20 female rats per group) at a ratio of one male rat: two female rats. Mating was confirmed by the presence of sperm in the vaginal smear and was considered as day 0.5 of gestation. Our earlier data on fertility studies in the GIO group have shown that WNIN/Ob male rats were infertile and did not produce any litters [10]. Thus, the resorbed and normal embryos were acquired only from the DIO group. Gravid female rats were separated and sacrificed at day 18.5 of gestation and the fertility parameters, namely, potency, litter size, number of implantation sites, corpora lutea, number of live, dead and resorbed embryos were assessed as reported earlier [10]. Resorbed and normal embryos were dissected, snap-frozen in liquid nitrogen and immediately stored at −80 °C for RNA extraction. The collected resorbed and normal embryos were sired by different male rats within the same group (n = 6 per group).

After the mating studies, the male rats from both the DIO and GIO groups were sacrificed and both the testes excised. One testis was snap-frozen in liquid nitrogen and stored at -80 °C for RNA extraction and the other testis was processed for flow cytometry studies. For the collection of caudal spermatozoa, both the cauda epididymides were excised in DMEM (Dulbecco's Modified Eagle Medium, pH—7.4) (Sigma, St Louis, MO, USA) and incubated at 37 °C for 30 min, allowing sperm to diffuse in the medium. The sperms were passed through a 40 µm cell strainer and washed with hypotonic solution containing 0.45% NaCl so as to remove any contaminating cells. The sperms were then washed with PBS (0.01 M phosphate buffer containing 0.154 M NaCl; pH 7.4) and stored at −80 °C for DNA extraction. A schematic representation of the experimental design is shown in Fig. 1.

### Genomic DNA extraction from spermatozoa

Genomic DNA was extracted from 10 million caudal spermatozoa (n = 10 animals per group) using GeneAll Exgene Cell SV mini kit (GeneAll Biotechnology, Seoul, South Korea) as per manufacturer’s instructions. Briefly, spermatozoa were lysed using H2 buffer containing 20 mM Tris HCl (pH-8.0), 20 mM EDTA, 200 mM NaCl, 4% SDS, 80 mM DTT and proteinase K and incubated at 56 °C for 1 h. The lysate was then subjected to RNase A treatment for 2 min followed by further lysis using BL buffer at 56 °C for 10 min. DNA was precipitated using chilled absolute ethanol, washed and eluted in nuclease-free water. Genomic DNA yield and purity were assessed using a UV spectrophotometer. Genomic DNA with the absorbance ratio 260/280 nm of 1.6—1.8 was further processed for global DNA methylation and methylation sequencing.

### Global DNA methylation analysis

Global DNA methylation levels of caudal spermatozoa were estimated using a 5-mC DNA ELISA kit (Zymoresearch, CA, USA) as per the manufacturer’s protocol. Briefly, 100 ng of genomic DNA (n = 10 animals per group) from all the five groups were diluted in coating buffer and denatured at 98 °C for 5 min in a thermal cycler followed by incubation on ice for 10 min. The denatured DNA was immobilized on the microtitre plate (in duplicate) and incubated at 37 °C for 1 h. This was followed by blocking with a 5-mC ELISA buffer at 37 °C for 30 min. A standard curve was generated of known 5-mC percentage by preparing the mixtures of negative control and positive control, provided in the kit. Antibody mixture containing anti-5-methylcytosine and secondary antibody in 5-mC ELISA buffer was added in all the wells and incubated at 37 °C for 1 h, followed by washing. The monoclonal antibody binds to 5-mC in single-stranded DNA, with no detectable cross-reactivity to non-methylated or hydoxymethylated cytosines. However, the antibody could detect methylated cytosines in any context and it was not sequence specific. The reaction was detected using an HRP developer followed by color development in 10 min at room temperature (RT). The absorbance was measured at 450 nm using ELISA plate reader. The global 5-mC content was calculated from the standard curve. Percent CpG methylation (%5mC/CpG sites) levels in spermatozoa were calculated by multiplying the observed optical density (OD) from the standard curve by the fold difference between E. coli (control DNA derives from E. coli) and the rat genome according to the manufacturer’s instructions.

### Combined 5-methylcytosine (5-mC) and propidium iodide (PI) staining using flow cytometry

Testicular cell-type-specific 5-mC content was determined using flow cytometry (protocol adapted from Desjobert et al. 2015 [67]). Testicular single-cell suspension (n = 6 animals per group) was prepared by passing the seminiferous tubules through 100 and 70 µm cell strainers. One million testicular cells were fixed using 4% paraformaldehyde for 15 min at RT. The cells are then washed with PBS, followed by permeabilization with 0.5% Triton X-100 prepared in PBS, for 15 min at RT. Cells were again washed and treated with 2 N HCl for 30 min at 37 °C. This was followed by neutralization with 100 mM Tris–HCl (pH-8.8) for 10 min at RT and extensive washes with 0.05% Tween 20 in PBS. The cells were then blocked for 2 h with 1% BSA and 0.05% Tween 20 in PBS and incubated either with a 5-mC antibody (host: mouse; 1:50; catalog no. A3001-50; Zymoresearch, CA, U.S.A.) or isotypic control antibody for 90 min at RT. This monoclonal antibody was both sensitive and specific for 5-mC in single-stranded DNA and showed no cross-reactivity to non-methylated or hydroxylated cytosines. It could detect methylated cytosines in any context and was not sequence specific. Cells were then washed with 1% BSA and 0.05% Tween 20 in PBS and incubated with secondary antibody conjugated to a fluorophore, for 45 min at RT. The samples were then washed twice with 1% BSA and 0.05% Tween 20 in PBS and stained with PI (5 µg/ml) for 2 h at 4 °C for flow cytometry analysis. Cell labeling was analyzed using FACS Aria SORP (Becton Dickinson; San Jose, CA, USA) and 10,000 ungated events were recorded. Flow cytometry data were analyzed using FACS Diva 6.1.3 software (BD, San Jose, CA, USA). Briefly, testicular cells were selected according to their forward scatter (FSC) and side scatter (SSC) parameters (P1 region) to exclude cell debris and then gated based on their PI content (P2 region) to exclude cell doublets, aggregates and apoptotic cells (Additional file 5—S3). 5-mC and PI fluorescence intensities of the gated cells (P2 region) were reported as dot plots and histograms, respectively (Additional file 5—S3). Dot plots display DNA methylation according to the DNA content (Additional file 5—S3). In order to compare the 5-mC density of the DNA of each testicular cell type, taking into consideration the difference in their DNA content, the mean fluorescence intensity (MFI) of 5-mC was combined with the MFI of PI (DNA) to calculate 5-mC/DNA index, as shown by Desjobert et al. 2015 [67]. Representative plots for CD versus HFD and LEAN versus CROb versus WNIN/Ob are shown as Additional 5: S4.

### Methylation sequencing (Methyl-Seq)

For high-throughput DNA methylation analysis in caudal spermatozoa (n = 3, pooled genomic DNA samples per group), methylation sequencing libraries were prepared with Illumina-compatible SureSelectXT Methyl-Seq Target Enrichment System for Illumina Multiplexed Sequencing (Agilent Technologies, Santa Clara, CA, USA) at Genotypic Technologies Pvt. Ltd., Bangalore, India. Briefly, approximately 1 μg of genomic DNA was sheared using Covaris S2 sonicator (Covaris, Woburn, Massachusetts, USA) to generate approximate fragment peak size between 150–200 bp. The fragment size distribution was checked on Agilent Bioanalyzer (Agilent Technologies, Palo Alto, CA, USA). The fragments were end-repaired, adenylated and ligated to Illumina multiplex barcode adaptors as per SureSelectXT Methyl-Seq Target Enrichment protocol. Adapter-ligated DNA was purified with HighPrep PCR clean up system. The ligated product was then used for hybridization as outlined in the SureSelectXT Methyl-Seq Target Enrichment protocol for Illumina Paired-End Sequencing Library manual using SureSelectXT Rat Methyl-Seq Reagent Kit. Hybridized library fragments were isolated by magnetic capture using Dynabeads™ MyOne™ Streptavidin T1 (Invitrogen, Carlsbad, CA, USA). The captured library was eluted in 20 μl of SureSelect Elution Buffer. The captured library was then subjected to bisulfite conversion using the EZ DNA Methylation Kit. Here, unmethylated cytosine residues in the library are converted to uracil residues and methylated cytosine residues remain unmodified. After desulphonation, the treated DNA was amplified by PCR, converting uracil residues in the sample to thymidine, using 8 PCR cycles. Clean up of PCR product was done using HighPrep PCR clean up system. The modified captured DNA library was indexed by PCR indexing amplification using 6 PCR cycles. The final PCR product (sequencing library) was purified with HighPrep PCR clean up system. Quantification and validation of the Captured Library were carried out using Qubit fluorometer and running an aliquot on Agilent Bioanalyzer. Finally, the sequencing library was accurately quantified by quantitative PCR using the Kapa Library Quantification Kit (Kapa Biosystems, Wilmington, MA, USA). The qPCR quantified libraries were pooled in equimolar amounts to create final equimolar amounts for multiplexed paired end sequencing on Illumina NextSeq 550 sequencer for 75 cycles.

### Sequencing data analysis

The Illumina paired end raw reads (~ 35 to ~ 40 million raw reads were generated for each sample) were quality checked using FastQC (https://www.bioinformatics.babraham.ac.uk/projects/download.html). The raw reads were processed using a PERL script developed at Genotypic Technologies Pvt. Ltd. for removal of adapters and low-quality bases. The processed data was mapped to the rat reference genome [Jul.2014 (RGSC 6.0/rn6)] using Bowtie2 version 2.2.7 [68] and methylated cytosines were identified using the Bismarkv0.16.3 tool [69]. The reference genome was converted into a bisulphite-converted version (conversion of C to T and G to A) and then indexed using Bowtie2-build program [68]. Methylation levels were extracted from the alignments using Bismarkv0.16.3 methylation extractor [69]. From alignment output, every C's which depends on the context as CpG, CHG and CHH (where H is A, C or T), the methylation call were extracted and reported using Bismarkv0.16.3 tool [69]. The percentage of methylation level was calculated by the number of methylated counts divided by all counts (methylated + non-methylated) summed across CpGs within the region. PCA plots were generated using MethylKit [70]. Methylated positions were annotated using Homer tool. Differentially methylated regions across samples were assessed using DESeq tool [71]. Log of global methylation data was retrieved from the DGE (differential gene expression) report and whisker box plots for global methylation of DMRs were developed using R script. Volcano plots were also created using the R script. Differences in fold coverage of DNA methylation were considered significant when the p value was < 0.05 and that the log2 foldchange > 1 was considered hypermethylated and log2 foldchange < 1 was considered hypomethylated. Gene ontology analysis and pathway analysis was done using PANTHER (PANTHER 14.1) and KEGG databases. A p value and false discovery rate (FDR) cutoff of < 0.05 were applied to obtain the list of significant pathways. Further, normalized methylation values from DGE were retrieved and heatmaps for each pathway were generated using Heatmap2 R package.

### Pyrosequencing

Validation of differentially methylated genes in spermatozoa was carried out using Pyrosequencing. Genomic DNA from spermatozoa (n = 5 per group) was subjected to bisulfite modification using MethylCode Bisulfite Conversion Kit (Invitrogen) as per manufacturer’s instructions. Briefly, 1.5 µg of genomic DNA was sequentially denaturated, sulphonated, deaminated and desulphonated to convert the unmethylated cytosines to uracil whereas the methylated cytosines remain unmodified. The modified DNA was eluted and used for PCR amplification of the genes of interest. PCR was performed using primers specific for modified sequences of genes of interest (Sigma, St Louis, MO, USA) using the Pyromark PCR amplification kit (Qiagen, Hilden, Germany). The primers were designed based on the coordinates for the point methylation change at the CpG site for the genes of interest obtained after methylation sequencing data analysis (Tables 1, 2). Either the forward or reverse primer was biotinylated. In order to check for completion of bisulfite reaction, the modified DNA was first subjected to PCR with primers specific for genomic DNA sequence (i.e., wild-set primers) of the same locus that is to be analyzed. A completely converted sample would not give any amplification with these primers. Genomic DNA was used as a template for positive control (Additional 5: S6). Primer sequences, coordinates, annealing temperature and amplicon sizes are listed in Additional file 1: S1.12 and Additional file 2—S2.11. Amplification conditions used were as follows: initial denaturation for 15 min at 95 °C, followed by 45 cycles of denaturation at 95 °C for 30 s, primer annealing for 30 s, extension at 72 °C for 30 s, and final extension at 72 °C for 10 min. The PCR products were bound to streptavidin-coated Sepharose beads (GE Healthcare Bio-Sciences AB, Uppsala, Sweden). Bound PCR products were denatured, and the non-biotinylated strand was washed off. The bound biotinylated strand was annealed to the sequencing primer, which was then subjected to pyrosequencing in PyroMark Q96 ID (Qiagen). Pyrosequencing detects the percentage of nucleotides at a given position in a sequence; therefore, it could be used to identify the level of C or T at a given CpG site. It was important to note that the PyroMark Q96 ID Pyrosequencer (Qiagen) used in the present study could detect only CpG methylation and not non-CpG methylation. The sequence of the template and the positions of the CpGs were marked and instructed before the run. At non-variable positions only the expected nucleotide was dispensed while at the variable position, i.e., of CpG, both C and T were dispensed and the amount of incorporated nucleotides revealed the proportion of C or T at the CpG position. The dispensation order was predetermined by the software for nucleotide incorporation. The nucleotides expected to be incorporated and nucleotides which were expected to give no signal were included (called blank dispensation). Each generated signal that was not associated with a CpG site (methylation detection site) is called a reference peak, used as internal control concerning quality issues and to calculate the expected single peak height. The histogram function provided by the software represents the theoretical peak height, which is used to validate the observed peak height. In-built internal controls and conversion controls were provided for DNA methylation analysis. As conversion control, thymines in the forward direction or adenines in the reverse direction were provided by the software to control the bisulfite-mediated reaction. Qiagen pyro Q-CpG software provided an efficient quality control system for each sequencing run. Conversion controls of completely bisulfite converted genomic DNA at a specific locus did not show any intensity signal. Default settings of the software for the threshold to pass quality control (blue assay) was a percentage of 4.5 and a percentage of 7.0 for conversion controls that have to be rechecked manually (yellow colored) [72].Representative pyrograms are shown as Additional 5: S7.

### RNA extraction and quantitative real-time PCR

Total RNA was extracted from 100 mg of the testis (n = 6, per group) and whole resorbed and normal embryos (n = 6, per group, day 18.5 of gestation) using TRI pure (Roche Diagnostics) as per the manufacturer’s instructions. Total RNA was treated with DNAse I enzyme at 37 °C for 1 h. RNA concentration and purity were assessed by measuring the absorbance at 260 and 280 nm. Total RNA (2 µg) was reverse transcribed using a High-Capacity cDNA reverse transcription kit from Applied Biosystems (Foster City, CA, USA) as per manufacturer’s protocol. Quantitative Real-time PCR was performed on Light cycler 96 Real-time PCR system (Roche) using Takyon SYBR green master mix (Eurogentec, Seraing, Belgium). The relative expression levels of genes of interest were estimated in relation to *Rn18s*. Amplification reactions (20 µl) containing 1.6 µl cDNA, 10 pM of respective primers and SYBR green master mix with the thermal cycling conditions of initial denaturation of 10 min at 95 °C followed by 40 cycles of 95 °C for 10 s, primer annealing temperature for 10 s, and extension at 72 °C for 10 s. Amplification reactions were run in duplicate and a no template control was included. Primer sequences, accession number, product size, and annealing temperature are mentioned in Additional file 1: S1.13. To check the specificity of the primers, melt curve analysis was performed and all the PCR products yielded the predicted melting temperature (Additional file 5—S2). Primer efficiencies were calculated using a standard curve (Additional file 1: S1.13). Pfaffl method was used to quantitate the relative gene expression. All the quantitative Real-time PCR procedures and data analysis followed MIQE guidelines (Additional file 1: S1.14).

### Statistical analysis

Data analysis was performed using Graph Pad Prism (version 5; Graph Pad Inc.; San Diego, CA, USA). For comparison between CD and HFD groups, unpaired Student's t-test was used with Welch’s correction. For comparison between LEAN, CROb, and WNIN/Ob groups, one way ANOVA was used. The level of significance was considered as p ≤ 0.05. All the data are represented as mean ± S.E.M.

## Supplementary information

**Additional file 1:** Detailed information on HFD group compared to CD group.

**Additional file 2:** Detailed information on WNIN/Ob group compared to CROb group.

**Additional file 3:** Detailed information on WNIN/Ob group compared to LEAN group.

**Additional file 4:** Detailed information on CROb group compared to LEAN group.

**Additional file 5:** Supplementary Figures S1–S13.

## Data Availability

Datasets analyzed during the current study will be made available from the corresponding author upon reasonable request. The supplementary data is available as additional files in the manuscript.

## Abbreviations

GIO: Genetically inherited obese/obesity
DIO: Diet-induced obese/obesity
qRT-PCR: Quantitative real-time polymerase chain reaction
CD: Control diet
HFD: High-fat diet
CROb: Calorie-restricted WNIN/Ob
5-mC: 5-Methylcytosine
ELISA: Enzyme-linked immunosorbent assay
ART: Assisted reproductive technology
RNA: Ribonucleic acid
POL: Post-implantation loss
DMEM: Dulbecco's modified eagle medium
NaCl: Sodium chloride
DNA: Deoxyribonucleic acid
Tris HCl: Tris hydrochloric acid
EDTA: Ethylenediaminetetraacetic acid
DTT: Dithiothreitol
RNase A: Ribonuclease A
UV spectrophotometer: Ultraviolet spectrophotometer
HRP developer: Horseradish peroxidase developer
PI: Propidium iodide
PBS: Phosphate buffer saline
RT: Room temperature
BSA: Bovine serum albumin
MFI: Mean fluorescence intensity
FSC: Forward scatter
SSC: Side scatter
PANTHER: Protein analysis through evolutionary relationships
KEGG: Kyoto Encyclopedia of Genes and Genomes
MIQE: Minimum Information for Publication of Quantitative Real-Time PCR Experiments
ANOVA: Analysis of variance
Dnmt: DNA methyltransferase
TGF-β: Transforming growth factor-β
SAM: S-adenosyl methionine
MAPK: Mitogen-activated protein kinase
JAK-STAT: Janus kinase (JAK)-signal transducer and activator of transcription (STAT)
PCP: Planar cell polarity
KO: Knockout
HAT: Histone acetyltransferase
HDAC: Histone deacetylase
HMT: Histone methyltransferase
HDM: Histone demethylases
Tet: Ten-eleven translocation
PCA: Principal component analysis
DGE: Differential gene expression

## References

[1] Palmer NO, Bakos HW, Fullston T, Lane M (2012). Impact of obesity on male fertility, sperm function and molecular composition. Spermatogenesis..

[2] Fullston T, Ohlsson Teague EM, Palmer NO, DeBlasio MJ, Mitchell M, Corbett M (2013). Paternal obesity initiates metabolic disturbances in two generations of mice with incomplete penetrance to the F2 generation and alters the transcriptional profile of testis and sperm microRNA content. FASEB J..

[3] Chambers TJG, Morgan MD, Heger AH, Sharpe RM, Drake AJ (2016). High-fat diet disrupts metabolism in two generations of rats in a parent-of-origin specific manner. Sci Rep..

[4] Zhang G, Pradhan S (2014). Mammalian epigenetic mechanisms. IUBMB Life.

[5] Berger SL, Sassone-Corsi P (2016). Metabolic Signaling to Chromatin. Cold Spring Harb Perspect Biol..

[6] de Castro BT, Ingerslev LR, Alm PS, Versteyhe S, Massart J, Rasmussen M (2015). High-fat diet reprograms the epigenome of rat spermatozoa and transgenerationally affects metabolism of the offspring. Mol Metab..

[7] Donkin I, Versteyhe S, Ingerslev LR, Qian K, Mechta M, Nordkap L (2016). Obesity and Bariatric Surgery Drive Epigenetic Variation of Spermatozoa in Humans. Cell Metab..

[8] Nammi S, Koka S, Chinnala KM, Boini KM (2004). Obesity: an overview on its current perspectives and treatment options. Nutr J..

[9] Lutz TA, Woods SC (2012). Overview of animal models of obesity. Curr Protoc Pharmacol..

[10] Deshpande SS, Nemani H, Pothani S, Khambata K, Kumar A, Kallamadi PR, Balasinor NH (2019). Genetically inherited obesity and high-fat diet-induced obesity differentially alter spermatogenesis in adult male rats. Endocrinology.

[11] Kalashikam RR, Battula KK, Kirlampalli V, Friedman JM, Nappanveettil G (2013). Obese locus in WNIN/obese rat maps on chromosome 5 upstream of leptin receptor. PLoS ONE.

[12] Giridharan N, Harishankar N, Satyavani M (1996). A new rat model for the study of obesity. Scand J Lab Anim Sci..

[13] Harishankar N, Kumar PU, Sesikeran B, Giridharan N (2011). Obesity associated pathophysiological & histological changes in WNIN obese mutant rats. Indian J Med Res..

[14] Deshpande SS, Nemani H, Pothani S, Balasinor NH (2019). Altered endocrine, cytokine signaling and oxidative stress: A plausible reason for differential changes in testicular cells in diet-induced and genetically-inherited - obesity in adult rats. Reprod Biol..

[15] Biermann K, Steger K (2007). Epigenetics in male germ cells. J Androl..

[16] Bestor TH (2000). The DNA methyltransferases of mammals. Hum Mol Genet..

[17] Jue K, Bestor TH, Trasler JM (1995). Regulated synthesis and localization of DNA methyltransferase during spermatogenesis. Biol Reprod..

[18] Hata K, Okano M, Lei H, Li E (2002). Dnmt3L cooperates with the Dnmt3 family of de novo DNA methyltransferases to establish maternal imprints in mice. Development..

[19] Trasler JM (2009). Epigenetics in spermatogenesis. Mol Cell Endocrinol..

[20] Xu HX, Qin JZ, Zhang KY, Zeng WX (2015). Dynamic expression profile of DNA methyltransferases in rat testis development. Pol J Vet Sci..

[21] Zamudio NM, Scott HS, Wolski K, Lo CY, Law C, Leong D, Kinkel SA, Chong S, Jolley D, Smyth GK, de Kretser D, Whitelaw E, O'Bryan MK (2011). DNMT3L is a regulator of X chromosome compaction and post-meiotic gene transcription. PLoS ONE.

[22] Vigueras-Villaseñor RM, Rojas-Castañeda JC, Chávez-Saldaña M (2011). Alterations in the spermatic function generated by obesity in rats. Acta Histochem..

[23] Kietzmann T, Petry A, Shvetsova A, Gerhold JM, Görlach A, The epigenetic Kietzmann T, Petry A, Shvetsova A, Gerhold JM, Görlach A, (2017). The epigenetic landscape related to reactive oxygen species formation in the cardiovascular system. Br J Pharmacol.

[24] Rui J, Deng S, Lebastchi J, Clark PL, Usmani-Brown S, Herold KC (2016). Methylation of insulin DNA in response to proinflammatory cytokines during the progression of autoimmune diabetes in NOD mice. Diabetologia.

[25] Zhou SS, Zhou YM, Li D, Lun YZ (2011). Dietary methyl-consuming compounds and metabolic syndrome. Hypertens Res..

[26] Dumasia K, Kumar A, Deshpande S, Balasinor NH (2017). Estrogen signaling, through estrogen receptor β, regulates DNA methylation and its machinery in male germ line in adult rats. Epigenetics..

[27] Efimova OA, Pendina AA, Tikhonov AV (2017). Genome-wide 5-hydroxymethylcytosine patterns in human spermatogenesis are associated with semen quality. Oncotarget..

[28] Tomizawa S, Kobayashi H, Watanabe T (2011). Dynamic stage-specific changes in imprinted differentially methylated regions during early mammalian development and prevalence of non-CpG methylation in oocytes. Development..

[29] Teerds KJ, de Rooij DG, Keijer J (2011). Functional relationship between obesity and male reproduction: from humans to animal models. Hum Reprod Update..

[30] Jeschke J, Collignon E, Fuks F (2015). DNA methylome profiling beyond promoters - taking an epigenetic snapshot of the breast tumor microenvironment. FEBS J..

[31] Qu Y, Siggens L, Cordeddu L, Gaidzik VI, Karlsson K, Bullinger L, Döhner K, Ekwall K, Lehmann S, Lennartsson A (2017). Cancer-specific changes in DNA methylation reveal aberrant silencing and activation of enhancers in leukemia. Blood.

[32] Stemmler MP, Hecht A, Kemler R (2005). E-cadherin intron 2 contains cis-regulatory elements essential for gene expression. Development..

[33] Thomas RM, Sai H, Wells AD (2012). Conserved intergenic elements and DNA methylation cooperate to regulate transcription at the il17 locus. J Biol Chem..

[34] Lu L, Zhu G, Zhang C, Deng Q, Katsaros D, Mayne ST (2012). Association of large noncoding RNA HOTAIR expression and its downstream intergenic CpG island methylation with survival in breast cancer. Breast Cancer Res Treat..

[35] Schlesinger F, Smith AD, Gingeras TR, Hannon GJ, Hodges E (2013). De novo DNA demethylation and noncoding transcription define active intergenic regulatory elements. Genome Res..

[36] Berger NA (2014). Obesity and cancer pathogenesis. Ann N Y Acad Sci..

[37] Lesch BJ, Tothova Z, Morgan EA, Liao Z, Bronson RT, Ebert BL, Page DC (2019). Intergenerational epigenetic inheritance of cancer susceptibility in mammals. Elife..

[38] Huang X, Liu G, Guo J, Su Z (2018). The PI3K/AKT pathway in obesity and type 2 diabetes. Int J Biol Sci..

[39] Bost F, Aouadi M, Caron L, Binétruy B (2005). The role of MAPKs in adipocyte differentiation and obesity. Biochimie.

[40] Zhang G, Sun Q, Liu C (2016). Influencing factors of thermogenic adipose tissue activity. Front Physiol..

[41] Gurzov EN, Stanley WJ, Pappas EG, Thomas HE, Gough DJ (2016). The JAK/STAT pathway in obesity and diabetes. FEBS J..

[42] Namkoong S, Cho CS, Semple I, Lee JH (2018). Autophagy dysregulation and obesity-associated pathologies. Mol Cells..

[43] Guo S (2014). Insulin signaling, resistance, and the metabolic syndrome: insights from mouse models into disease mechanisms. J Endocrinol..

[44] Yang Y (2012). Wnt signaling in development and disease. Cell Biosci..

[45] Komiya Y, Habas R (2008). Wnt signal transduction pathways. Organogenesis..

[46] Carballo GB, Honorato JR, de Lopes GPF (2018). Spohr TCLSE. A highlight on Sonic hedgehog pathway. Cell Commun Signal.

[47] Armas-López L, Zúñiga J, Arrieta O, Ávila-Moreno F (2017). The Hedgehog-GLI pathway in embryonic development and cancer: implications for pulmonary oncology therapy. Oncotarget..

[48] Wu MY, Hill CS (2009). Tgf-beta superfamily signaling in embryonic development and homeostasis. Dev Cell..

[49] Wharton K, Derynck R (2009). TGFbeta family signaling: novel insights in development and disease. Development..

[50] Kopan R, Ilagan MX (2009). The canonical Notch signaling pathway: unfolding the activation mechanism. Cell.

[51] Kopan R (2012). Notch signaling. Cold Spring Harb Perspect Biol..

[52] van Amerongen R, Berns A (2006). Knockout mouse models to study Wnt signal transduction. Trends Genet..

[53] Pan YB, Gong Y, Ruan HF, Pan LY, Wu XK, Tang C (2015). Sonic hedgehog through Gli2 and Gli3 is required for the proper development of placental labyrinth. Cell Death Dis.

[54] Zhou J, Chen Q, Lanske B, Fleming BC, Terek R, Wei X (2014). Disrupting the Indian hedgehog signaling pathway in vivo attenuates surgically induced osteoarthritis progression in Col2a1-CreERT2; Ihhfl/fl mice. Arthritis Res Ther.

[55] Sasai N, Toriyama M, Kondo T (2019). Hedgehog Signal and Genetic Disorders. Front Genet.

[56] Oshima M, Oshima H, Taketo MM (1996). TGF-beta receptor type II deficiency results in defects of yolk sac hematopoiesis and vasculogenesis. Dev Biol..

[57] Attisano L, Lee-Hoeflich ST (2001). The smads. Genome Biol.

[58] Mašek J, Andersson ER (2017). The developmental biology of genetic Notch disorders. Development.

[59] Champroux A, Cocquet J, Henry-Berger J, Drevet JR, Kocer A (2018). A decade of exploring the mammalian sperm epigenome: paternal epigenetic and transgenerational inheritance. Front Cell Dev Biol..

[60] Pathak S, Saxena M, D'Souza R, Balasinor NH (2010). Disrupted imprinting status at the H19 differentially methylated region is associated with the resorbed embryo phenotype in rats. Reprod Fertil Dev..

[61] Seisenberger S, Peat JR, Hore TA, Santos F, Dean W, Reik W (2013). Reprogramming DNA methylation in the mammalian life cycle: building and breaking epigenetic barriers. Philos Trans R Soc Lond B Biol Sci..

[62] Shi L, Wu J (2009). Epigenetic regulation in mammalian preimplantation embryo development. Reprod Biol Endocrinol..

[63] Stobdan T, Sahoo D, Azad P (2019). High fat diet induces sex-specific differential gene expression in Drosophila melanogaster. PLoS ONE.

[64] Jang HS, Shin WJ, Lee JE, Do JT (2017). CpG and non-CpG methylation in epigenetic gene regulation and brain function. Genes (Basel)..

[65] Dubuc PU, Cahn PJ, Willis P (1984). The effects of exercise and food restriction on obesity and diabetes in young ob/ob mice. Int J Obes..

[66] Levin N, Nelson C, Gurney A, Vandlen R, de Sauvage F (1996). Decreased food intake does not completely account for adiposity reduction after ob protein infusion. Proc Natl Acad Sci USA.

[67] Desjobert C, El Maï M, Gérard-Hirne T (2015). Combined analysis of DNA methylation and cell cycle in cancer cells. Epigenetics..

[68] Langmead B, Salzberg SL (2012). Fast gapped-read alignment with Bowtie 2. Nat Methods..

[69] Krueger F, Andrews SR (2011). Bismark: a flexible aligner and methylation caller for Bisulfite-Seq applications. Bioinformatics.

[70] Akalin A, Kormaksson M, Li S (2012). methylKit: a comprehensive R package for the analysis of genome-wide DNA methylation profiles. Genome Biol..

[71] Anders S, Huber W (2010). Differential expression analysis for sequence count data. Genome Biol..

[72] Roessler J, Lehmann U (2015). Quantitative DNA Methylation Analysis by Pyrosequencing®. Methods Mol Biol..

