# Supplementary material for: High-fat diet-induced and genetically inherited obesity differentially alters DNA methylation profile in the germline of adult male rats
Source: Clin Epigenetics. 2020 Nov 19;12:179. doi: 10.1186/s13148-020-00974-7 (PMC7678167; doi:10.1186/s13148-020-00974-7)
Supplement: Supplementary file 5 — Additional file 5: Supplementary Figures S1–S13. [file 13148_2020_974_MOESM5_ESM.docx]

**Supplementary method:**

**Histological examination of the testis**

The testes (N=3 per group) were dissected and fixed in paraformaldehyde (PFA) for 24 hours. After primary fixation, the testis was cut into 3 pieces (3-5 mm thick) and refixed in a fresh fixative for 24 hours. The tissue was subjected different grades of alcohol, cleared in xylene and then embedded in paraffin wax. The paraffin embedded tissue were sectioned at a 5 μm thicknes, mounted on a poly lysine coated glass slide and then stained with Hematoxylin and Eosin (H & E) and observed under Ziess Axioskop photomicroscope at 20X magnification.

**
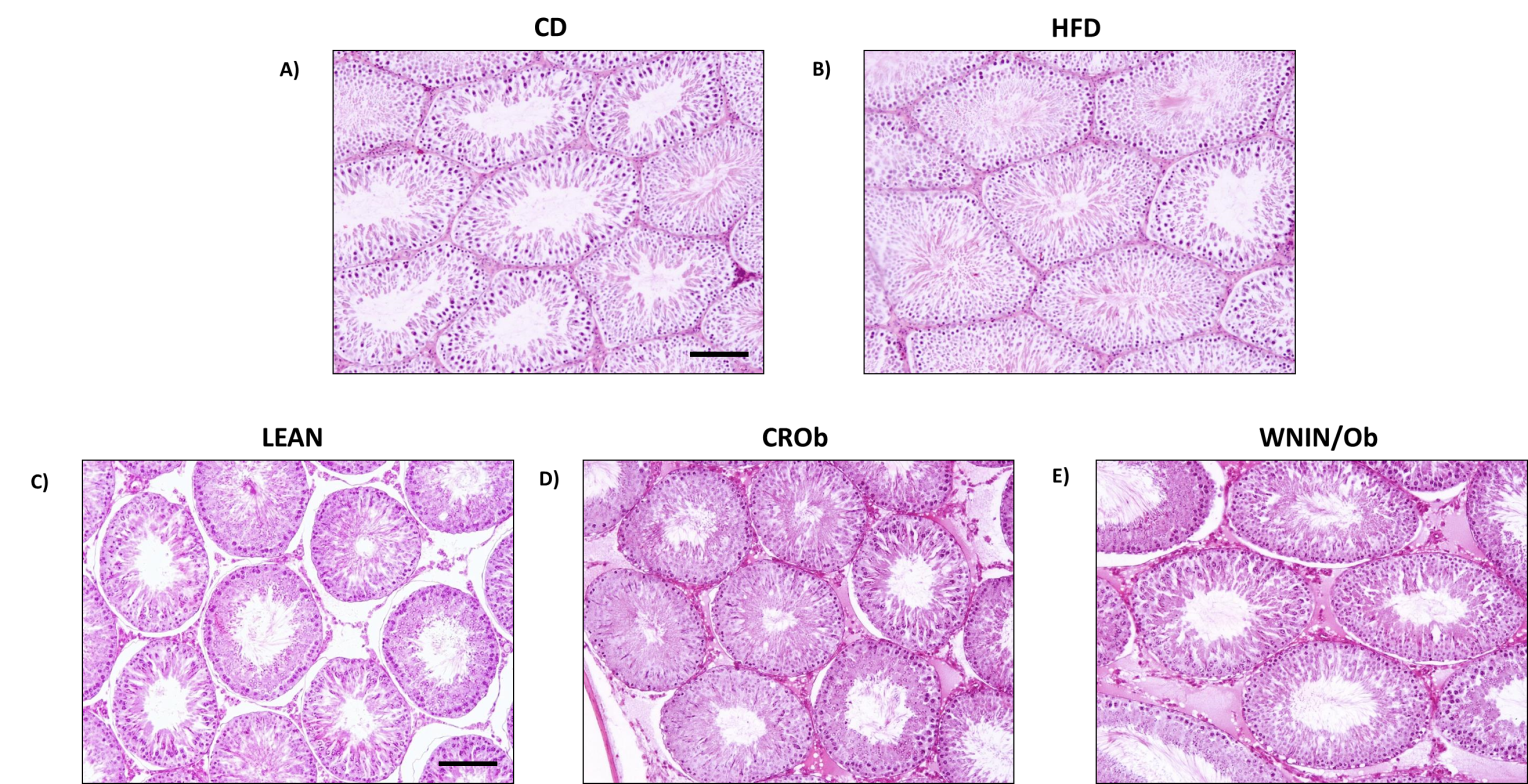
**

**Figure S1: Representative haematoxylin and eosin stained 5μm testicular sections showing seminiferous tubules from A) control diet (CD) group; B) high fat diet (HFD) group; C) LEAN group; D) calorie restricted WNIN/Ob (CROb) group and D) WNIN/Ob group. Scale bar represents 20 μm, N = 3 per group.**

**
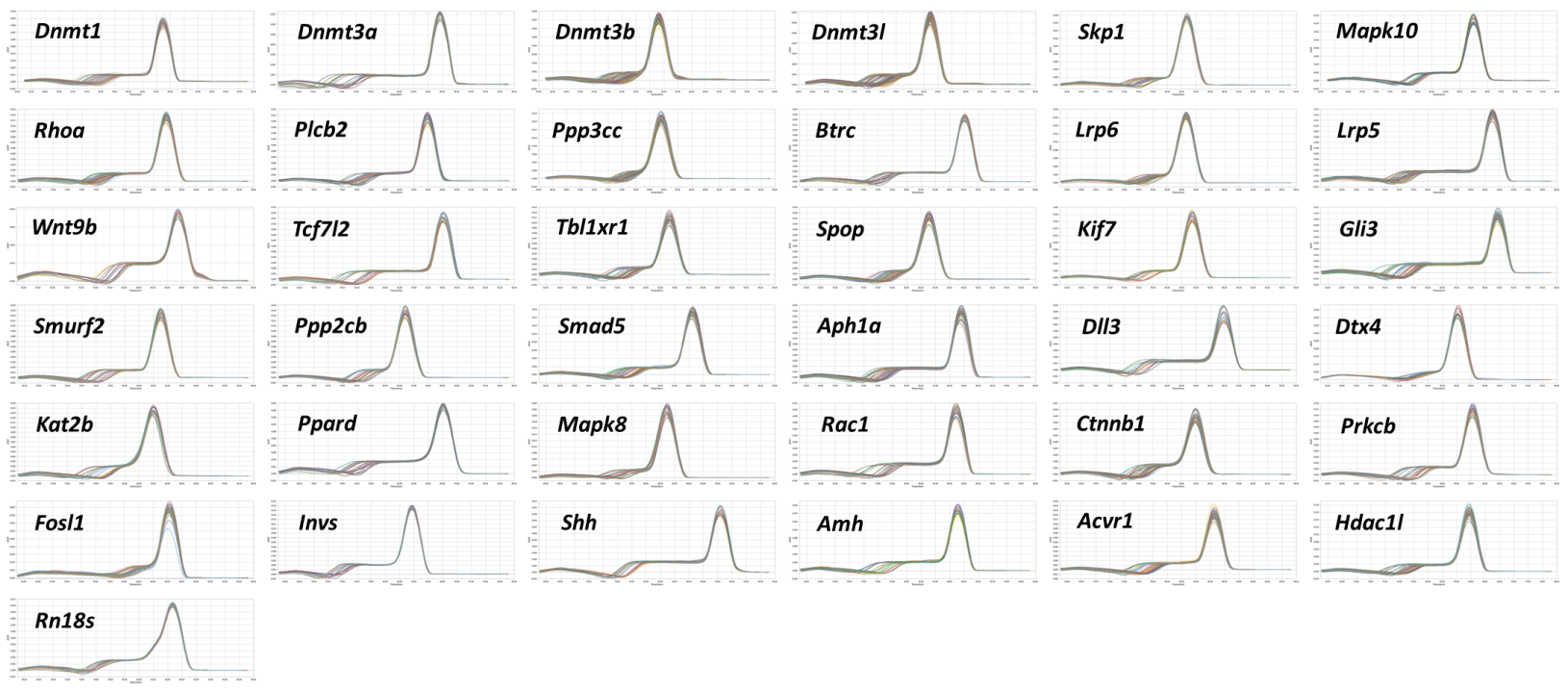
**

**Figure S2: Melt curves for genes analysed by qRT-PCR.**


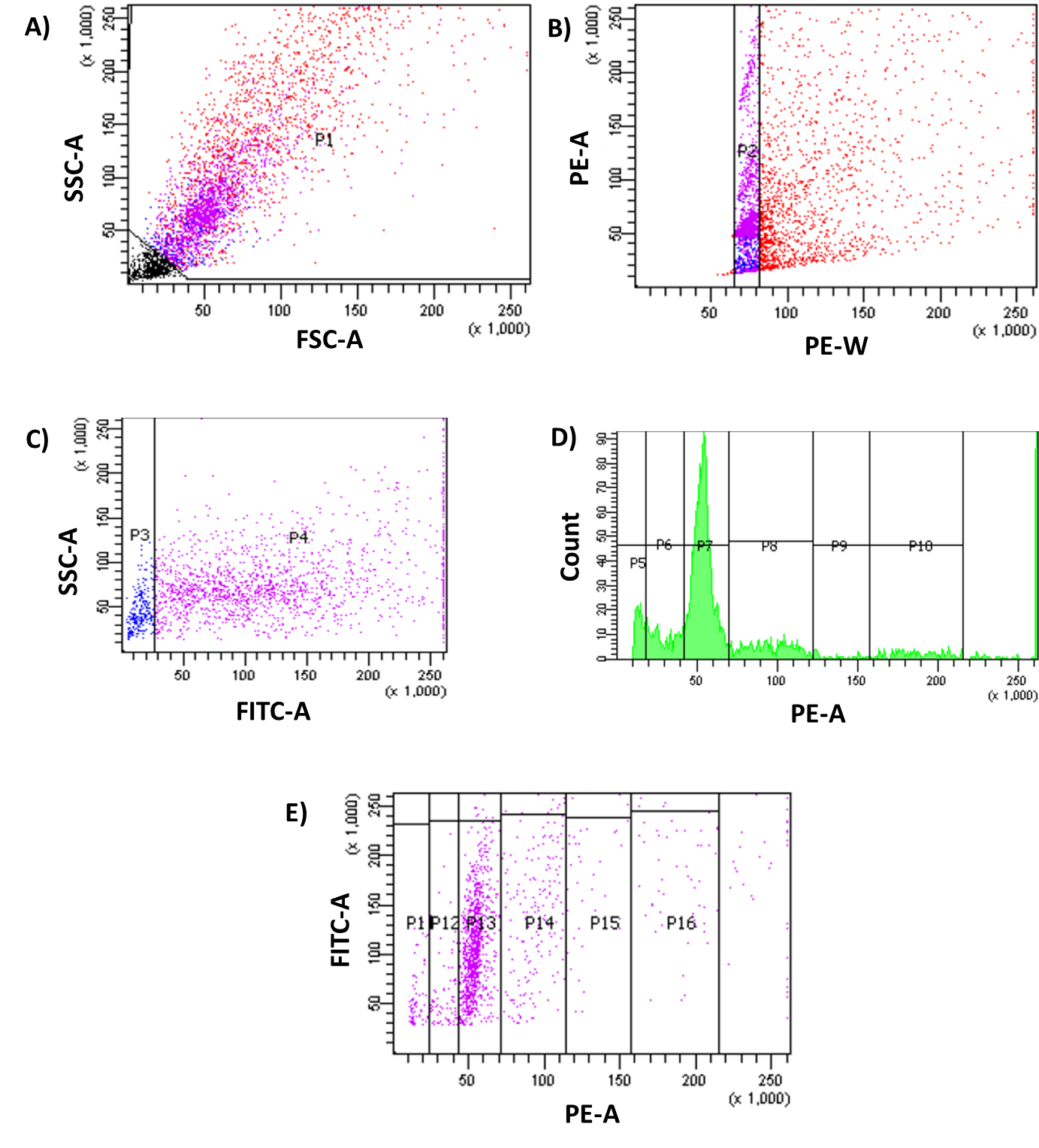


**Figure S3: Gating strategy for combined 5-methylcytosine (5-mC) and propidium iodide (PI) staining using flow cytometry. Testicular cells were labelled with anti-5-mC monoclonal antibody prior to DNA staining with propidium iodide (PI). For flow cytometry analysis, the testicular cells were selected A) according to their FSC and SSC parameters (P1 region) and B) then gated on their PI content (P2 region). C) 5mC labelling of the P2 cells (P4 purple region) and its isotypic control (P3 blue region) are displayed as a dot plot with a linear scale. Analysis of cell cycle (D) is combined with analysis of DNA methylation (C) and is displayed as a dot plot (E). P5 and P11 represents elongated spermatid population; P6 and P12 represents elongating spermatid population; P7 and P13 represents round spermatid population; P8 and P14 represents 2N population; P9 and P15 represents S phase population and P10 and P16 represents 4N population.**

**
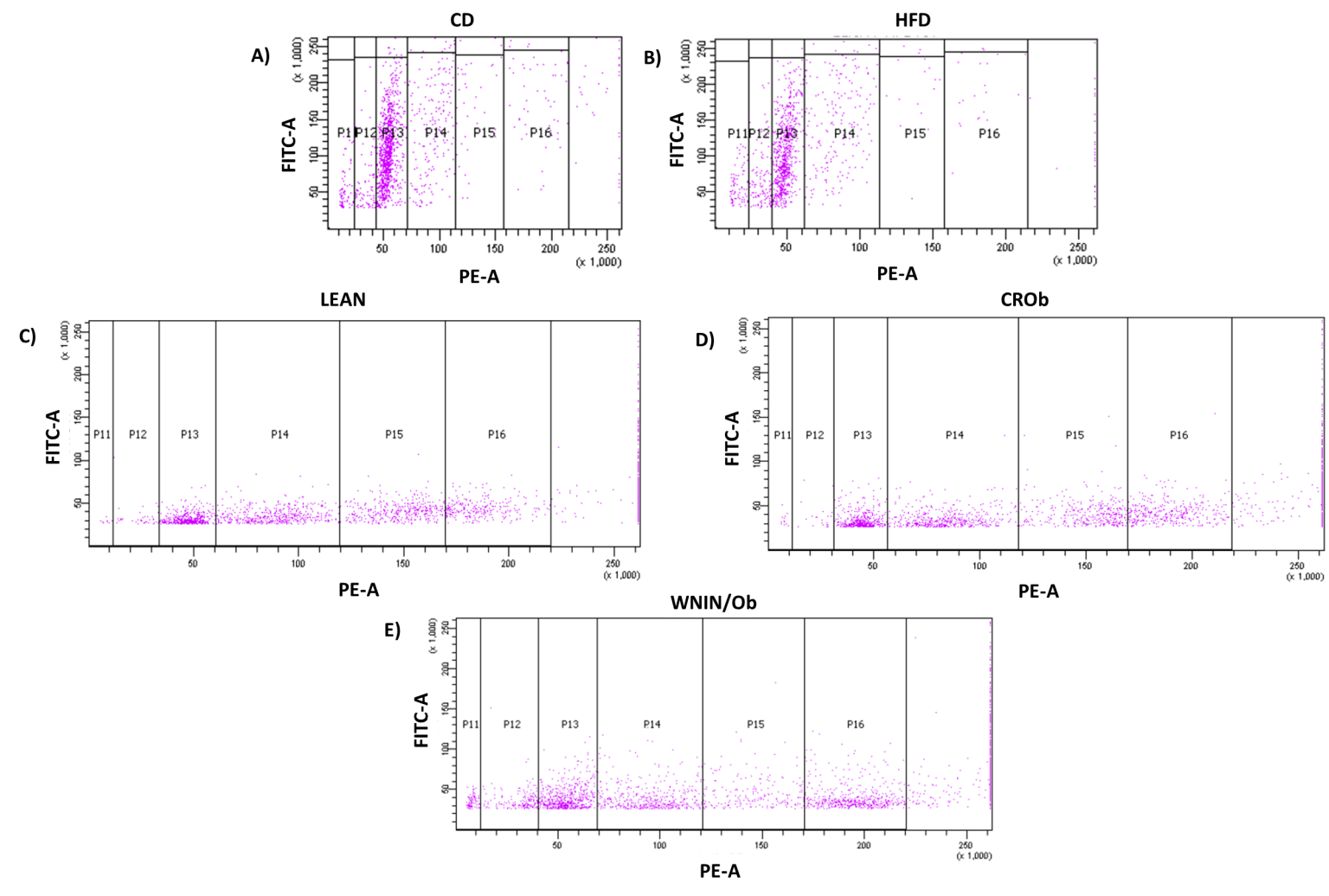
**

**Figure S4: Representative dot plots for combined 5-methylcytosine (5-mC) and propidium iodide (PI) staining using flow cytometry in the testis of CD vs HFD (A and B) and LEAN vs CROb vs WNIN/Ob (C, D and E) groups respectively. P5 and P11 represents elongated spermatid population; P6 and P12 represents elongating spermatid population; P7 and P13 represents round spermatid population; P8 and P14 represents 2N population; P9 and P15 represents S phase population and P10 and P16 represents 4N population.**

**
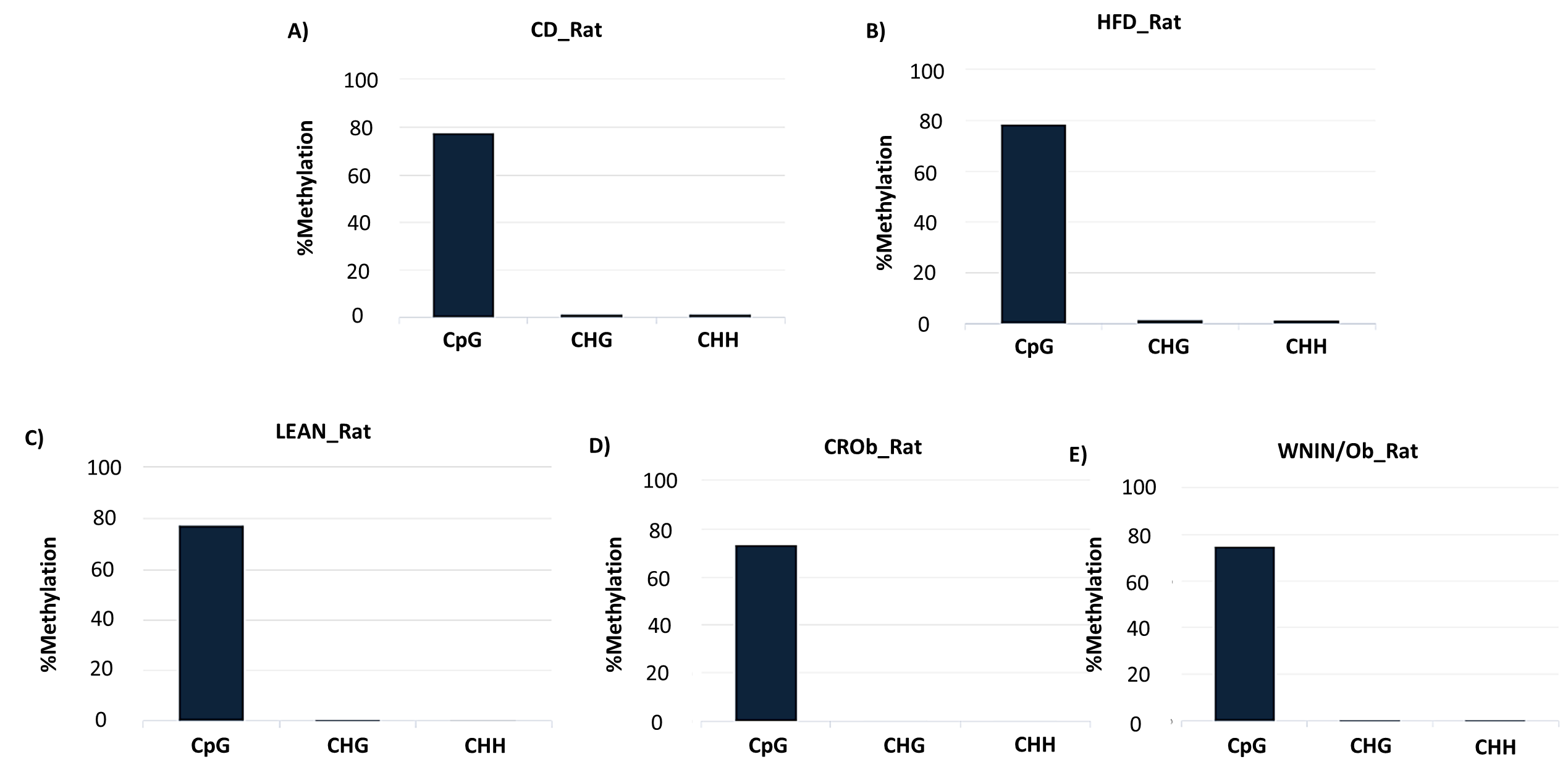
**

**Figure S5: Percentage methylation in CpG, CHG and CHH context for A) CD, B) HFD, C) LEAN, D) CROb and E) WNIN/Ob groups.**

**
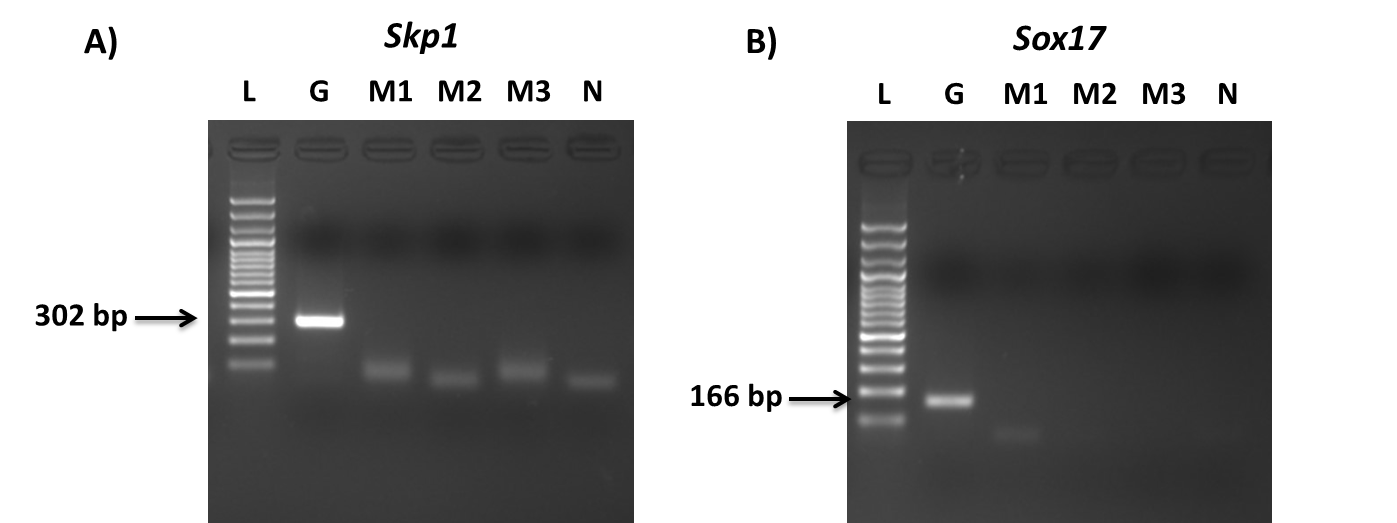
**

**Figure S6: PCR products obtained for A) *Skp1* and B) *Sox17*. ‘L’ represents 100 bp ladder; ‘G’ represents the genomic DNA; ‘M’ represents bisulfite modified DNA samples and ‘N’ represents no template control.**

**
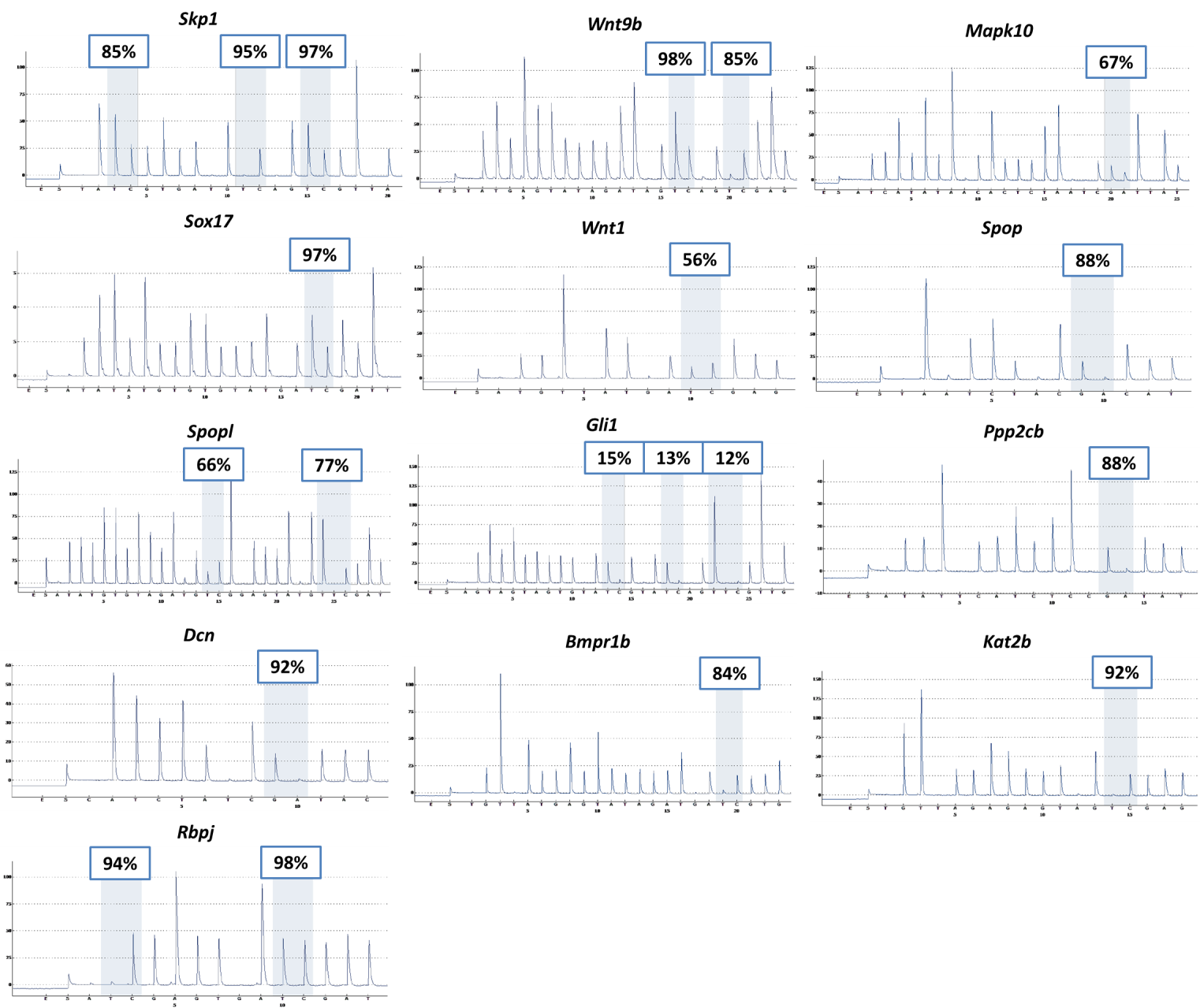
**

**Figure S7: Representative pyrograms of the differentially methylated genes validation by Pyrosequencing.**


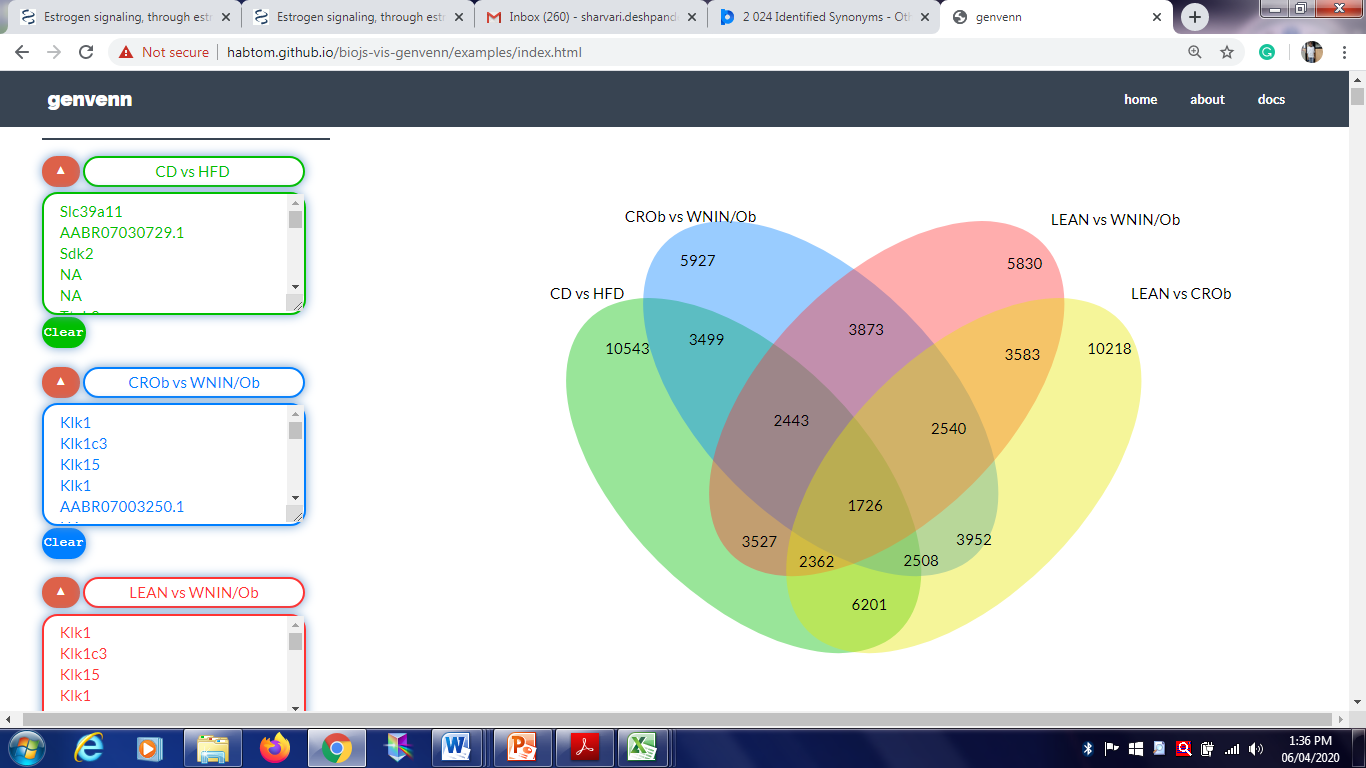


**Figure S8: Venn diagram representing the number of unique and overlapping differentially methylated genes in the spermatozoa of the HFD group compared to the CD group, WNIN/Ob group compared to the LEAN group and CROb group compared to the LEAN group respectively.**


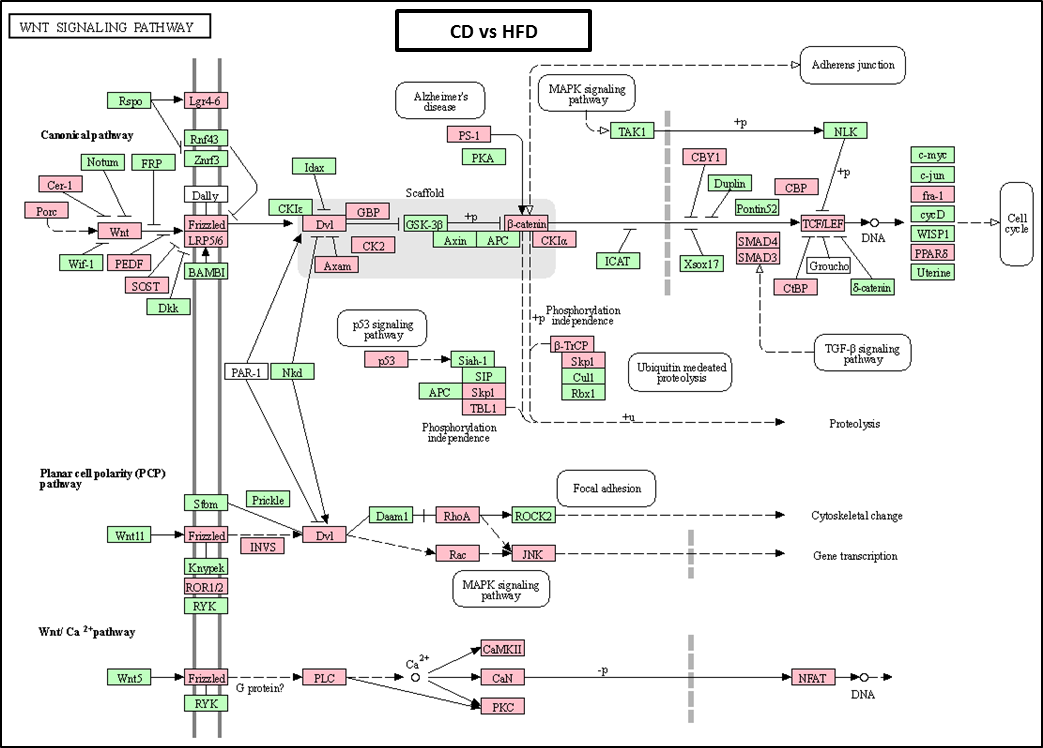


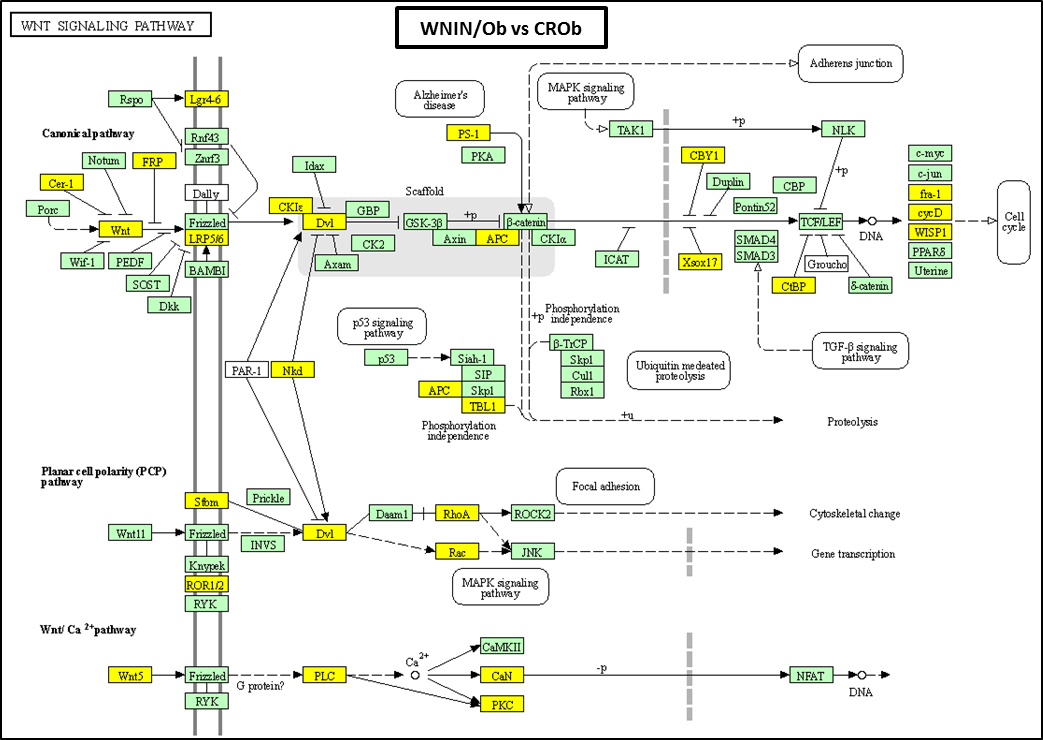


**Figure S9:** **Wnt signaling pathway: Figure illustrating genes involved in signaling of the Wnt pathway (KEGG database). Pink and yellow boxes indicate differentially methylated genes obtained from Methylation Sequencing in spermatozoa in the HFD group compared to the CD group and in the WNIN/Ob group compared to the CROb group respectively.**


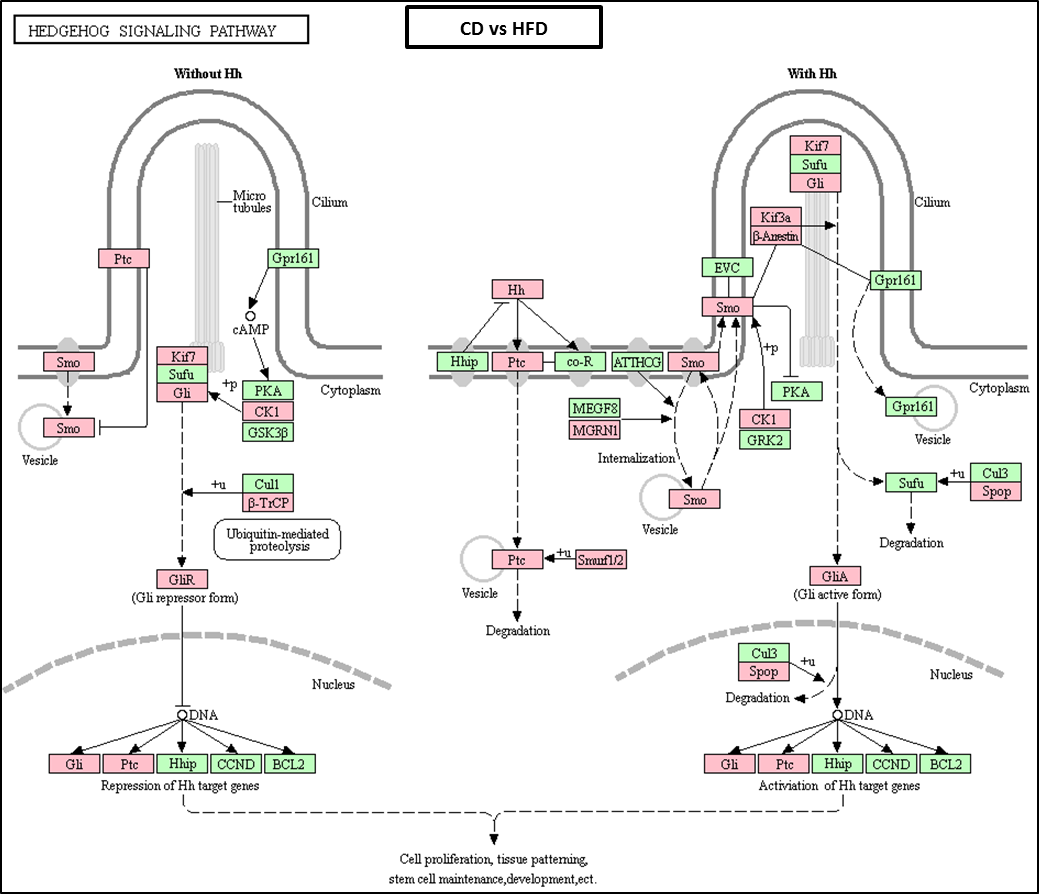


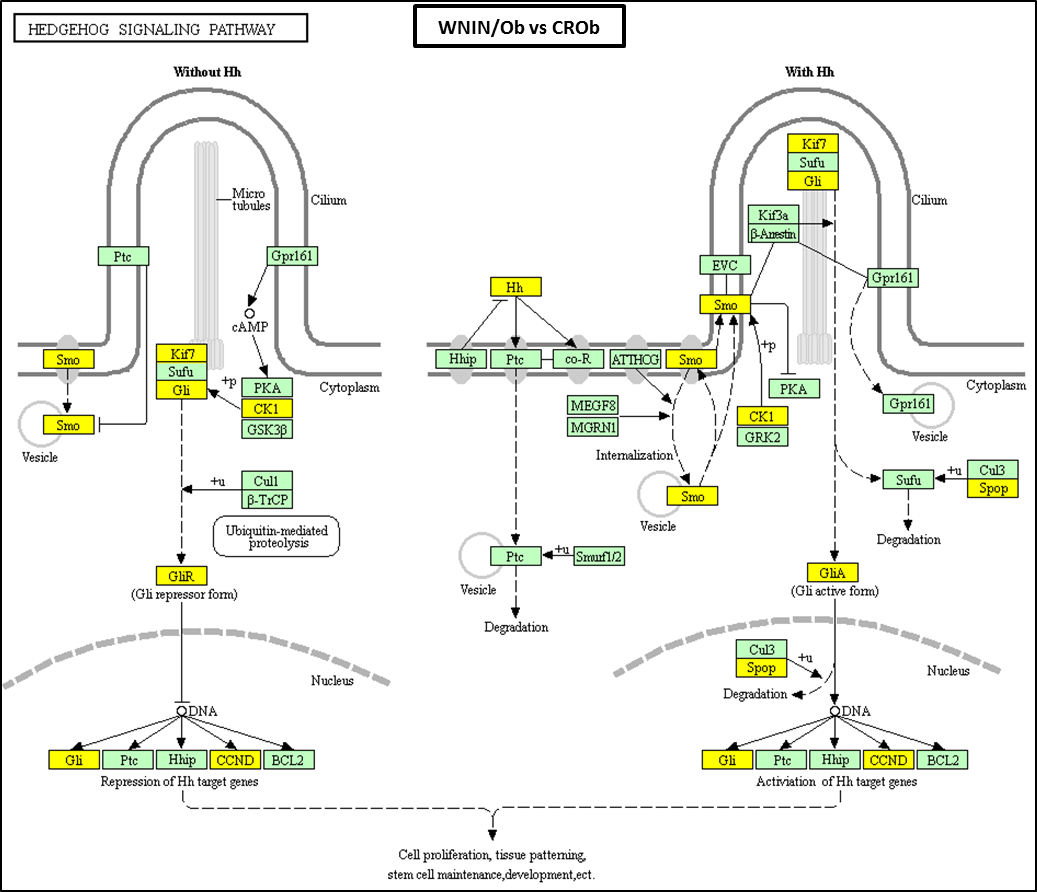


**Figure S10:** **Hedgehog signaling pathway: Figure illustrating genes involved in signaling of the Hedgehog pathway (KEGG database). Pink and yellow boxes indicate differentially methylated genes obtained from Methylation Sequencing in spermatozoa in the HFD group compared to the CD group and in the WNIN/Ob group compared to the CROb group respectively.**


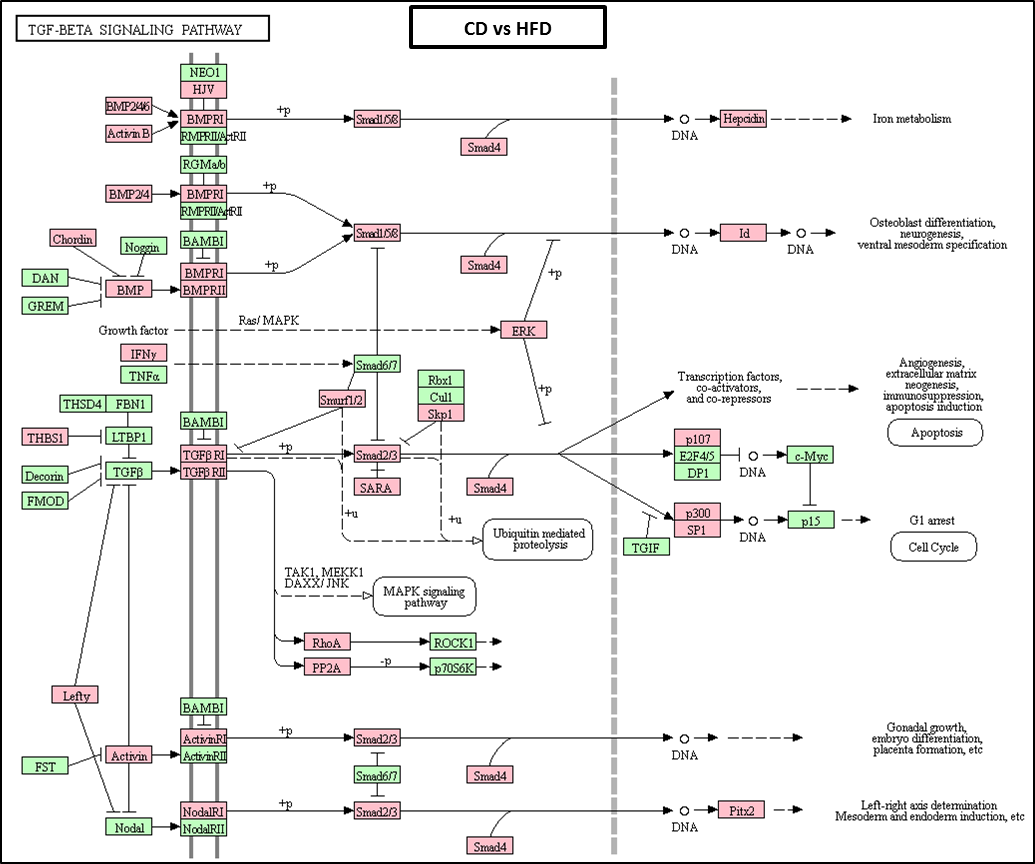


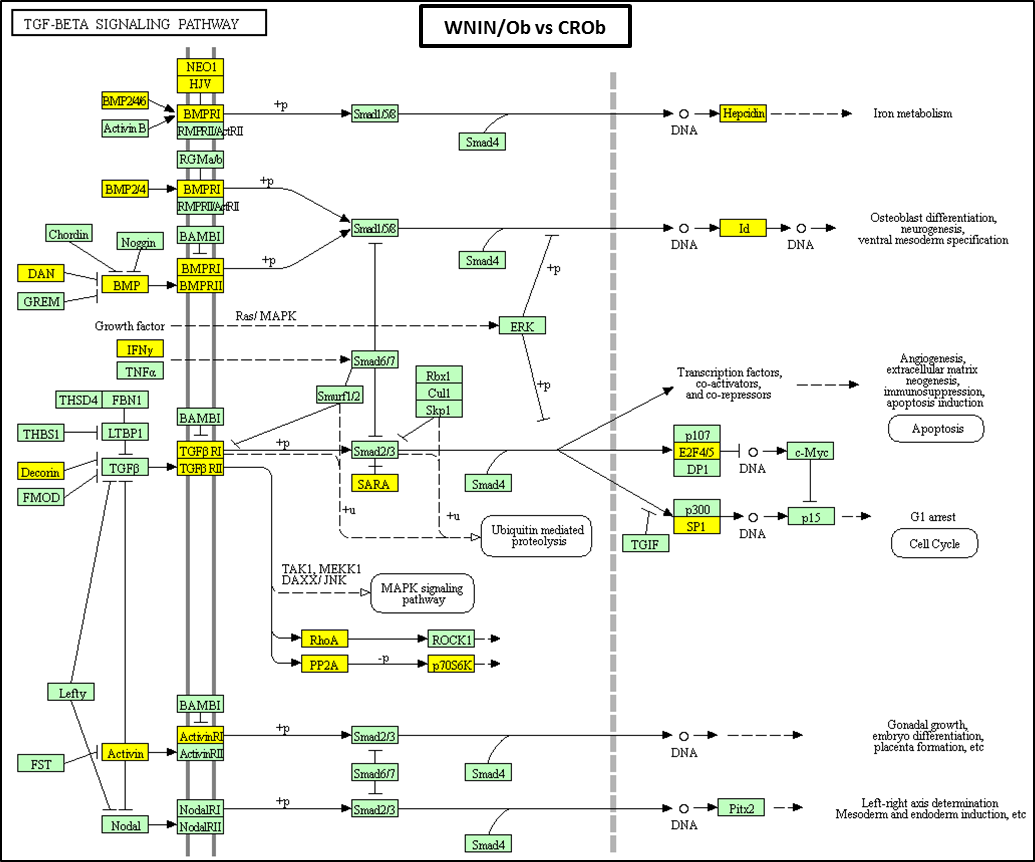


**Figure S11: TGF-beta signaling pathway: Figure illustrating genes involved in signaling of the TGF-beta pathway (KEGG database). Pink and yellow boxes indicate differentially methylated genes obtained from Methylation Sequencing in spermatozoa in the HFD group compared to the CD group and in the WNIN/Ob group compared to the CROb group respectively.**


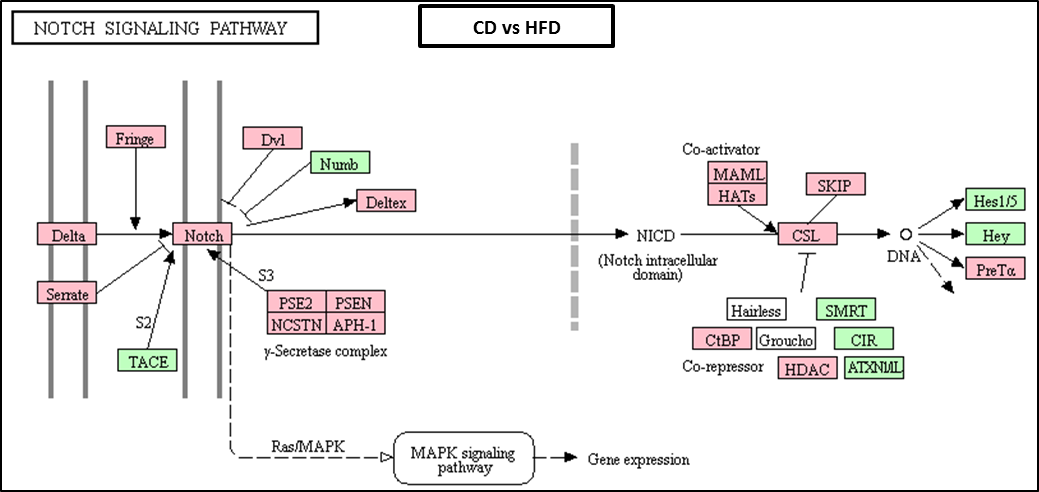


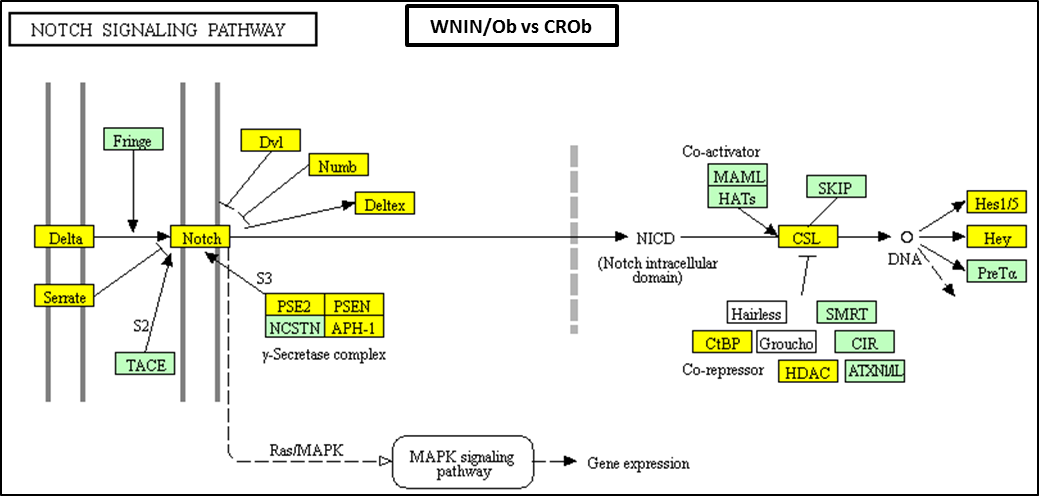


**Figure S12: Notch signaling pathway: Figure illustrating genes involved in signaling of the Notch pathway (KEGG database). Pink and yellow boxes indicate differentially methylated genes obtained from Methylation Sequencing in spermatozoa in the HFD group compared to the CD group and in the WNIN/Ob group compared to the CROb group respectively.**


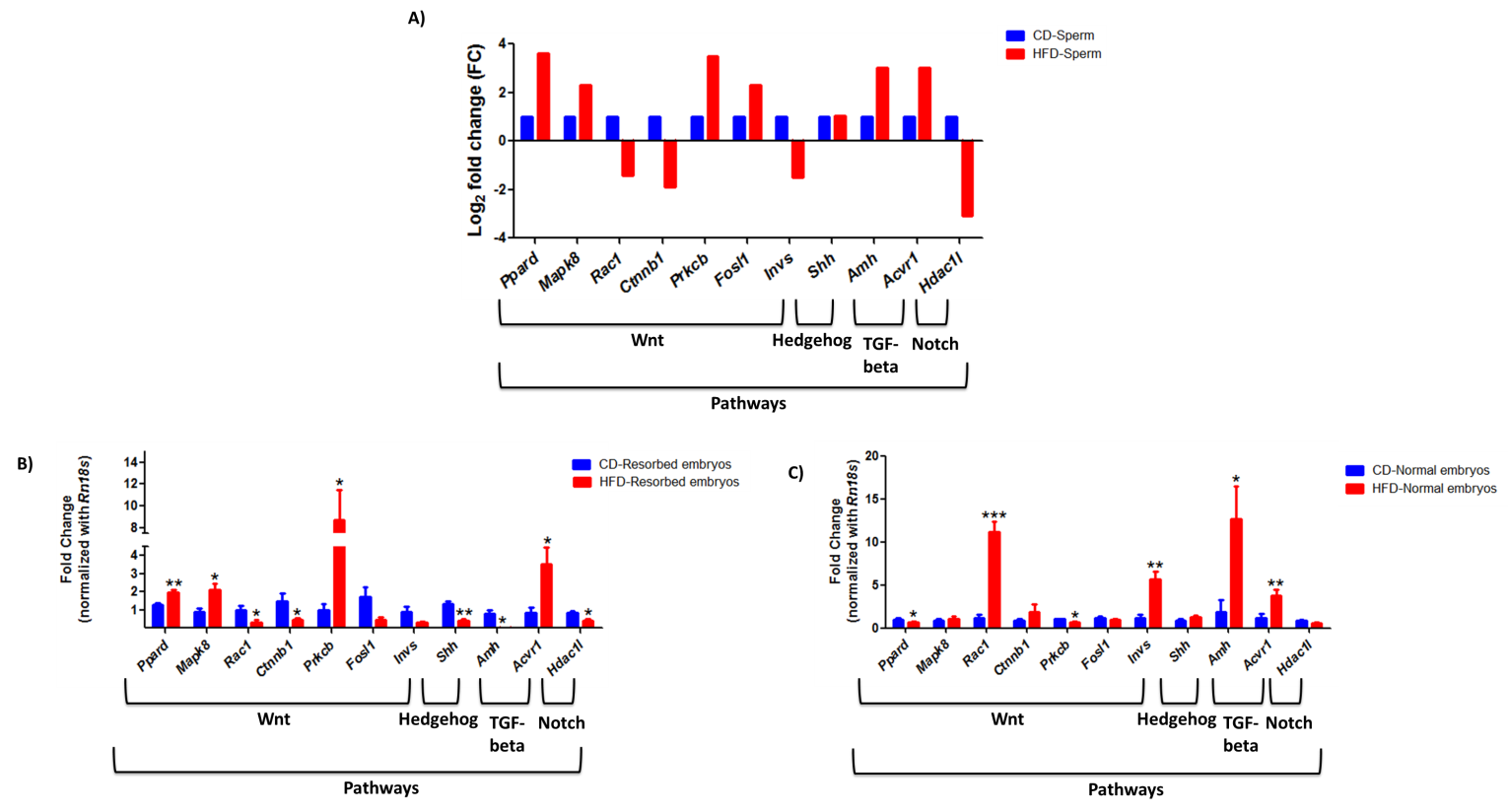


**Figure S13: Effect of high-fat diet-induced obesity on A) other differentially methylated genes enriched in Wnt, Hedgehog, TGF-beta, and Notch pathway in the spermatozoa. B) Gene expression pattern in the resorbed and normal embryos sired by diet-induced obese male rats. Data are expressed as means ± S.E.M, N=3 samples pooled per group for methylation sequencing and N=6 per group for gene expression studies. Asterisks indicate significant differences compared with the CD group (*p<0.05, **p<0.01).**
